# Olefin Coupling Catalyzed by (Pybox)Os Complexes via Osmacyclopentane Intermediates: Comparison with Isoelectronic (Phebox)Ir

**DOI:** 10.1021/jacsau.5c01197

**Published:** 2025-12-09

**Authors:** Ashish Parihar, Santanu Malakar, Soham Chakraborty, Michael C. Gallo, Thomas J. Emge, Faraj Hasanayn, Alan S. Goldman

**Affiliations:** a Department of Chemistry and Chemical Biology, Rutgers, The State University of New Jersey, New Brunswick, New Jersey 08901, United States; b Department of Chemistry, Rutgers University−Camden, Camden, New Jersey 08102, United States; c Department of Chemistry, 11238 American University of Beirut, Beirut 1107 2020, Lebanon

**Keywords:** alkene hydrovinylation, olefin dimerization, osmium catalysis, metallacyclopentane, pincer complexes, C−H activation, C−H elimination

## Abstract

(Pybox)Os is found to catalyze alkene hydrovinylation, effecting the dimerization of ethylene, tail-to-tail coupling of propene and 1-butene, and cross-coupling of ethylene with higher α-olefins. This reactivity contrasts with the previously reported dehydrogenative coupling of ethylene to give butadiene catalyzed by the isoelectronic fragment (Phebox)­Ir. The reaction mechanism was investigated through computational and experimental means. Both the Os- and Ir-catalyzed reactions proceed through a [2 + 2 + 1] cyclization of the corresponding bis-olefin complex to yield an experimentally observed metallacyclopentane intermediate. In both cases, the metallacyclopentane undergoes β-H elimination, via a dechelated κ^2^-pincer-ligated intermediate, to yield a σ–π-but-3-enyl hydride complex or derivative. Both the greater reactivity and the distinct chemoselectivity of the Os system relative to the Ir system are attributable to C–H reductive elimination by the σ–π-but-3-enyl hydride having a barrier for Os much lower than that for Ir. This lower barrier to C–H elimination for Os is unexpected given that the thermodynamic driving force for elimination is much less for Os than for Ir. Computational studies of model complexes were conducted, comparing (Pybox)­Os­(L)­(CH_3_)­(H) with the isoelectronic (Phebox)­Ir­(L)­(CH_3_)­(H). The results indicate that the more facile kinetics with Os relative to Ir may be general for C–H elimination from six-coordinate d^6^ complexes of the two metals, as well as for the microscopic reverse, i.e., C–H addition to the corresponding four-coordinate d^8^ species.

## Graphical abstract

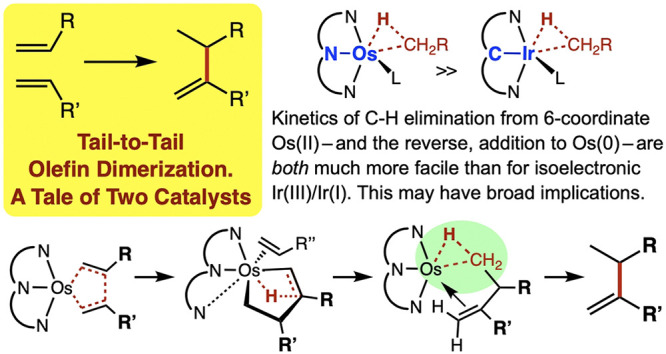

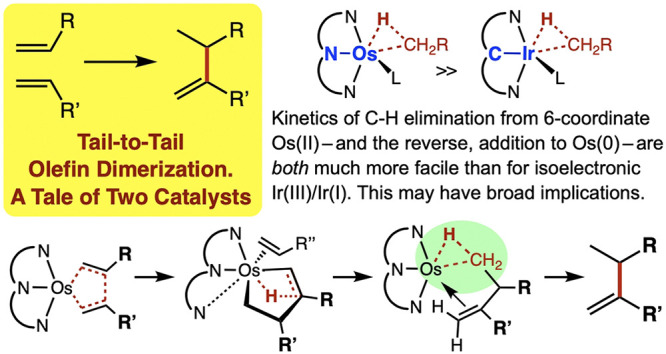

## Introduction

Olefin dimerization, or more generally coupling or hydrovinylation, is a reaction of great industrial importance.^1–15^ Coupling of linear α-olefins is of particular interest.^16^ Three different acyclic skeletal isomeric products are generally possible, head-to-tail (*h-t*), head-to-head (*h-h*), and tail-to-tail (*t-t*). Most commonly, coupling proceeds via the insertion of an olefin double bond into a M–H bond, the insertion of a second olefin into the resulting M–C bond (Cossee–Arlman reaction), and then β-H elimination; this generally yields *h-t* or *h-h* products. The *t-t* products, however, can be desirable for the higher degree of branching (and therefore higher octane numbers for gasoline)^5^ or as intermediates for organic synthesis.^3,17,18^ Although uncommon, reports of *t-t* product formation are not unprecedented^18,19^ and date back at least to Schrock’s seminal 1978 report of *t-t* propylene dimerization, proceeding via a metallacyclopentane.^19–21^

Several years ago, we reported that (Phebox)Ir catalyzed the formation of butadiene from ethylene (Scheme 1).^22^ This reaction was demonstrated to proceed via formation of an iridacyclopentane, which underwent β-H elimination to give a but-3-enyl iridium hydride. The but-3-enyl group ultimately underwent a second β-H elimination to yield butadiene. Somewhat disappointingly, the catalyst was not effective with higher olefins. In an effort to explore and extend this chemistry, we have investigated the isoelectronic (Pybox)Os analogue. This complex has proven to be a more active catalyst for coupling, including with higher olefins. Unlike the Ir catalyst, it yields dimers (monoenes) rather than dehydrogenative coupling products. Experimental and computational studies have been conducted to elucidate the mechanistic differences and origins of the different chemoselectivities of the two catalysts. The results indicate unanticipated, fundamental, reactivity differences between these congeners, with possible implications extending well beyond olefin coupling.^23^

**Scheme 1:**
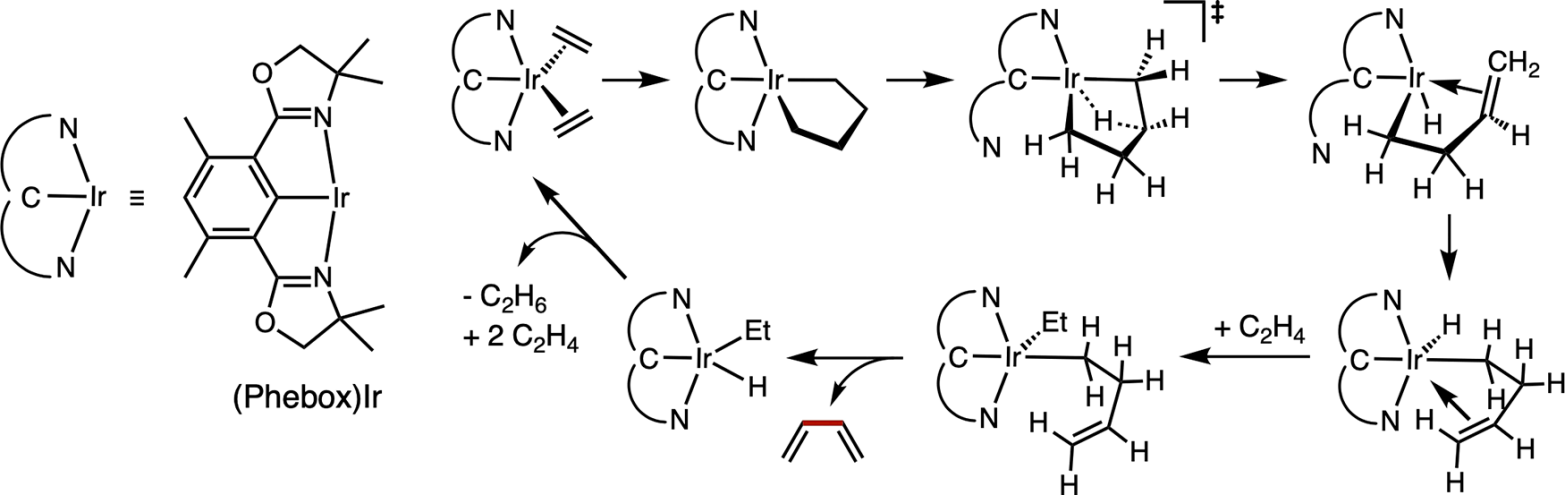
Dehydrogenative Coupling of Ethylene to Butadiene Catalyzed by (Phebox)­Ir (from Ref 22)

## Results and Discussion

### Synthesis of (Pybox)­OsCl_3_ (**1-Cl_3_**)

2,6-Bis­(4,4-dimethyl-4,5-dihydrooxazol-2-yl)­pyridine (Pybox)^24–26^ was allowed to react with (NH_4_)_2_OsCl_6_, K_2_OsCl_6_, or Na_2_OsCl_6_ at 120 °C for 2 days in 2-methoxyethanol, as reported previously^27^ for the reaction with ^tBu^PNP (2,6-bis­(di-*t*-butylphosphinomethyl)­pyridine) to yield (^tBu^PNP)­OsCl_3_. (Pybox)­OsCl_3_ (**1-Cl**_**3**_) was obtained in all cases (Figure 1a); presumably, the Os­(IV) precursors undergo reduction by the alcohol solvent.^28,29^ The ^1^H NMR spectrum (Figure S3) of **1-Cl**_**3**_ displayed far upfield and far downfield signals (details in the SI), characteristic of a paramagnetic complex.

**Figure 1:**
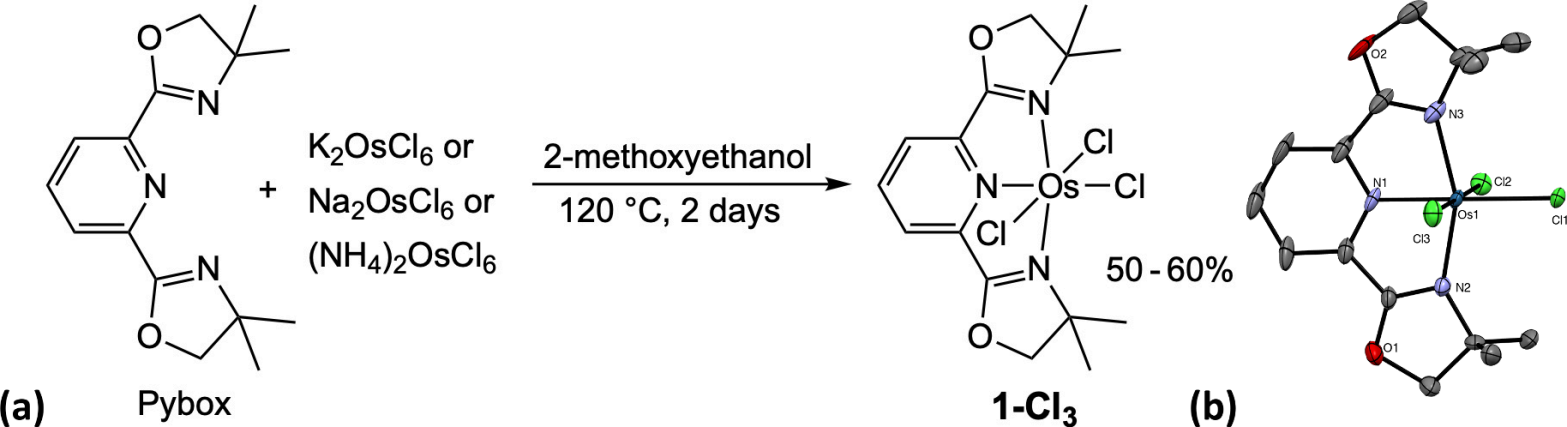
(a) Metalation of Pybox to generate **1-Cl**_3_. (b) Thermal ellipsoid plot (50% probability ellipsoids) of the structure of **1-Cl**_**3**_ determined by X-ray diffraction; CH_2_Cl_2_ solvate and H atoms omitted for clarity.

Crystals suitable for X-ray diffraction were obtained by slow evaporation of diethyl ether into a dichloromethane solution of **1-Cl**_**3**_. The molecular structure obtained was approximately octahedral at osmium (Figure 1b).

### Synthesis of [(Pybox)­OsH_3_^–^] (**1-H_3_** ^**–**^)

In an attempt to synthesize the tetrahydride complex, **1-H**_**4**_ (by analogy with our previously reported^27^ synthesis of (^tBu^PNP)­OsH_4_), 1 equiv **1-Cl**_**3**_ was treated with 4 equiv KO^t^Bu under 1 atm H_2_ in THF for 24 h (Scheme 2). A single upfield peak (−13.2 ppm) was observed in the ^1^H NMR spectrum integrating to 3H relative to the Pybox ligand signals. Crystals were obtained by vapor diffusion of pentane into a THF solution at room temperature. Single-crystal X-ray diffractometry revealed a dimeric structure, with (Pybox)­OsH_3_^–^ units bridged by 2 K^+^. The hydrides were located with a relatively high degree of confidence (see the SI). Each K^+^ cation was bound to a N=C–C=N linkage of a Pybox ligand and a hydride of the same unit (*d*_KH_ = 2.9 Å), while also bridging the partner unit via interaction with two of its hydrides (*d*_KH_ ∼ 2.8 Å) (Figure 2). Surprisingly, the geometry at the Os centers was very distorted from octahedral. Of particular note, H^t^–Os–H^b1^ angles were quite acute, 58° and 53°, while the H^t^–Os–N^py^ angles were distinctly obtuse (ca. 118°) despite the fact that the position of H^t^ is not constrained by bridging with K^+^.

**Scheme 2:**
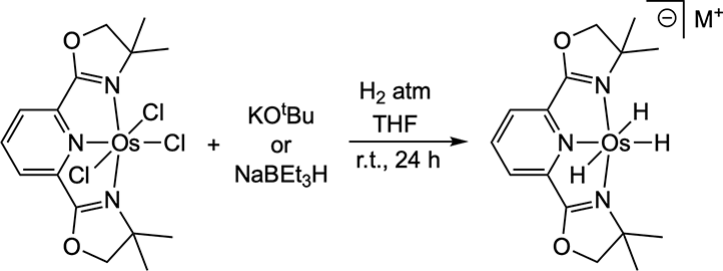
Reaction of **1-Cl_3_** with KO*^t^* Bu or NaBEt_3_H under a H_2_ Atmosphere to Generate [1-H_3_^–^]

**Figure 2:**
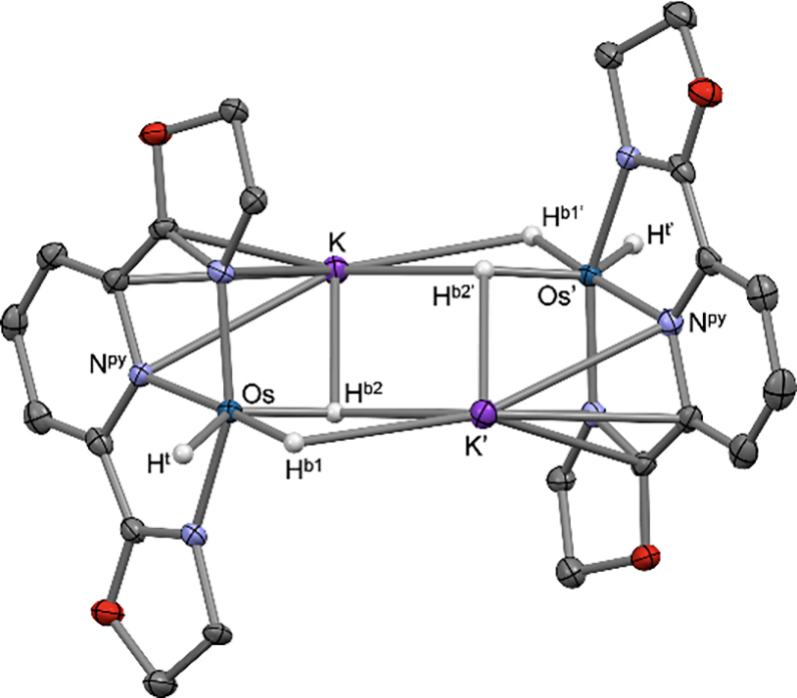
Thermal ellipsoid plot of the structure of **[1-H**_**3**_ **]**_**2**_ **[K­(THF)**_**2**_ **]**_**2**_. H atoms (except for hydride ligands), oxazoline methyl groups, and THF solvate molecules have been omitted for clarity.

**1-H**_**3**_^**–**^ could also be generated by the reaction of **1-Cl**_**3**_ with 4 equiv of NaBEt_3_H under 1 atm of H_2_ in THF for 24 h. Precipitation by the addition of pentane to the THF solution at ca. −40 °C led to the formation of what was presumed to be Na^+^[**1-H**_**3**_^**–**^], which appeared pure by ^1^H NMR spectroscopy (−14.0 ppm). The results of catalysis and reactivity experiments described below gave results independent of which route was used to generate **1-H**_**3**_^**–**^ or the presumed nature of the cation.

### Catalytic Olefin Homocoupling

Addition of ethylene to a benzene solution of K^+^[**1-H**_**3**_^**–**^] (4 mM) results in complete conversion to a new complex after ca. 12 h at room temperature or ca. 50 min at 80 °C. The product was identified by 1D and COSY ^1^H NMR, ^13^C NMR, and ^1^H–^13^C gHSQC spectroscopy, as an osmacyclopentane ethylene complex (**2-C**_**2**_ **H**_**4**_; Scheme 3), in analogy with the isoelectronic (Phebox)Ir metallacyclopentane ethylene complex that we have previously reported. To account for formation of **2-C**_**2**_ **H**_**4**_, we presume that a proton was obtained from a source carried over from the synthesis,^30^ such as ^t^BuOH, or perhaps from adventitious moisture.

**Scheme 3:**
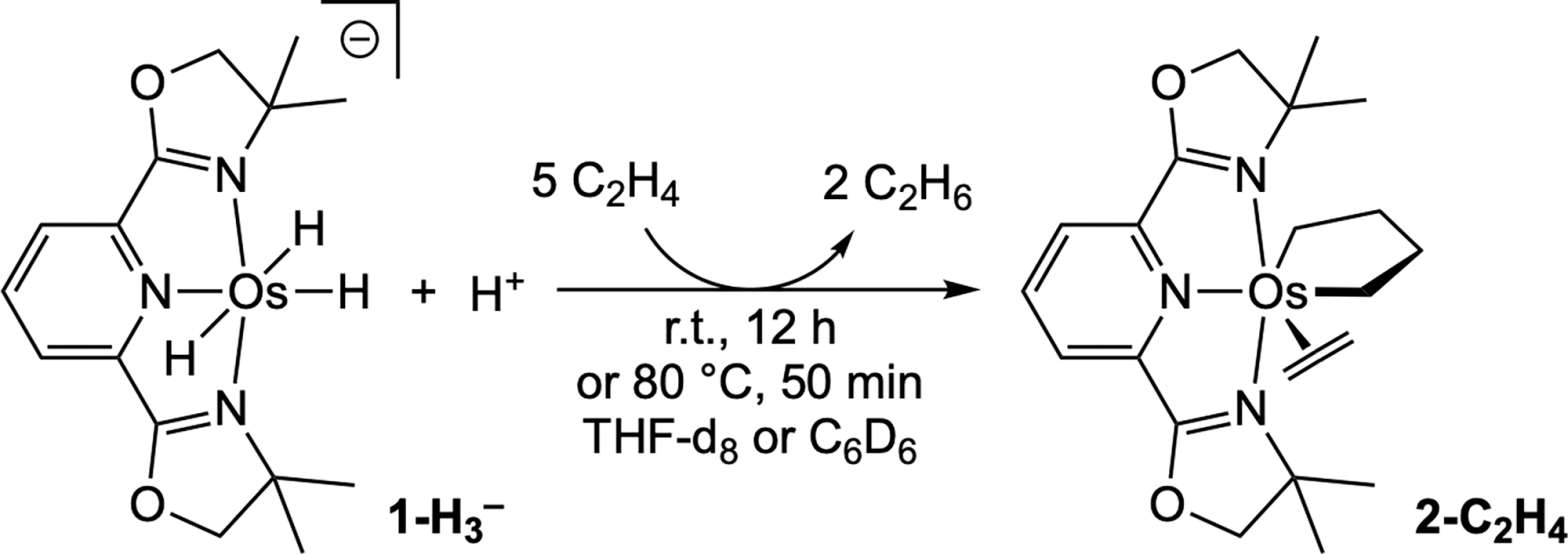
Formation of **2-C_2_H_4_** from the Reaction of [**1-H_3_^–^**] under C_2_H_4_ (1 atm)

Removal of volatiles from solutions of **2-C**_**2**_ **H**_**4**_ resulted in decomposition; replacement of solvent resulted in multiple species as indicated by ^1^H NMR spectroscopy. Addition of PMe_3_ (ca. 6 equiv) to a fresh THF-*d*_8_ solution of **2-C**_**2**_ **H**_**4**_ resulted in substitution to yield **2-PMe**_**3**_ as determined by ^1^H and ^31^P­{^1^H} NMR. Removal of volatiles from this solution yielded an amorphous solid which could be redissolved in THF-*d*_8_ without a change in the ^1^H or ^31^P­{^1^H} NMR spectra of **2-PMe**_**3**_, and the complex was further characterized by COSY ^1^H NMR, ^13^C NMR, and ^1^H–^13^C gHSQC spectroscopy (see SI).

After generating a solution of **2-C**_**2**_ **H**_**4**_ in situ (Scheme 3), further heating at 80 °C for 18 h under ethylene (1 atm) results in ca. 70% conversion to olefin dimerization products 1-, *cis*-, and *trans*-2-butene (50 TO, Scheme 4). At very early times and low conversion (ca. 3 mM), 1-butene was the predominant product, but over time, the mixture became predominantly 2-butenes, presumably reflecting isomerization and the thermodynamic equilibrium between isomers.

**Scheme 4:**
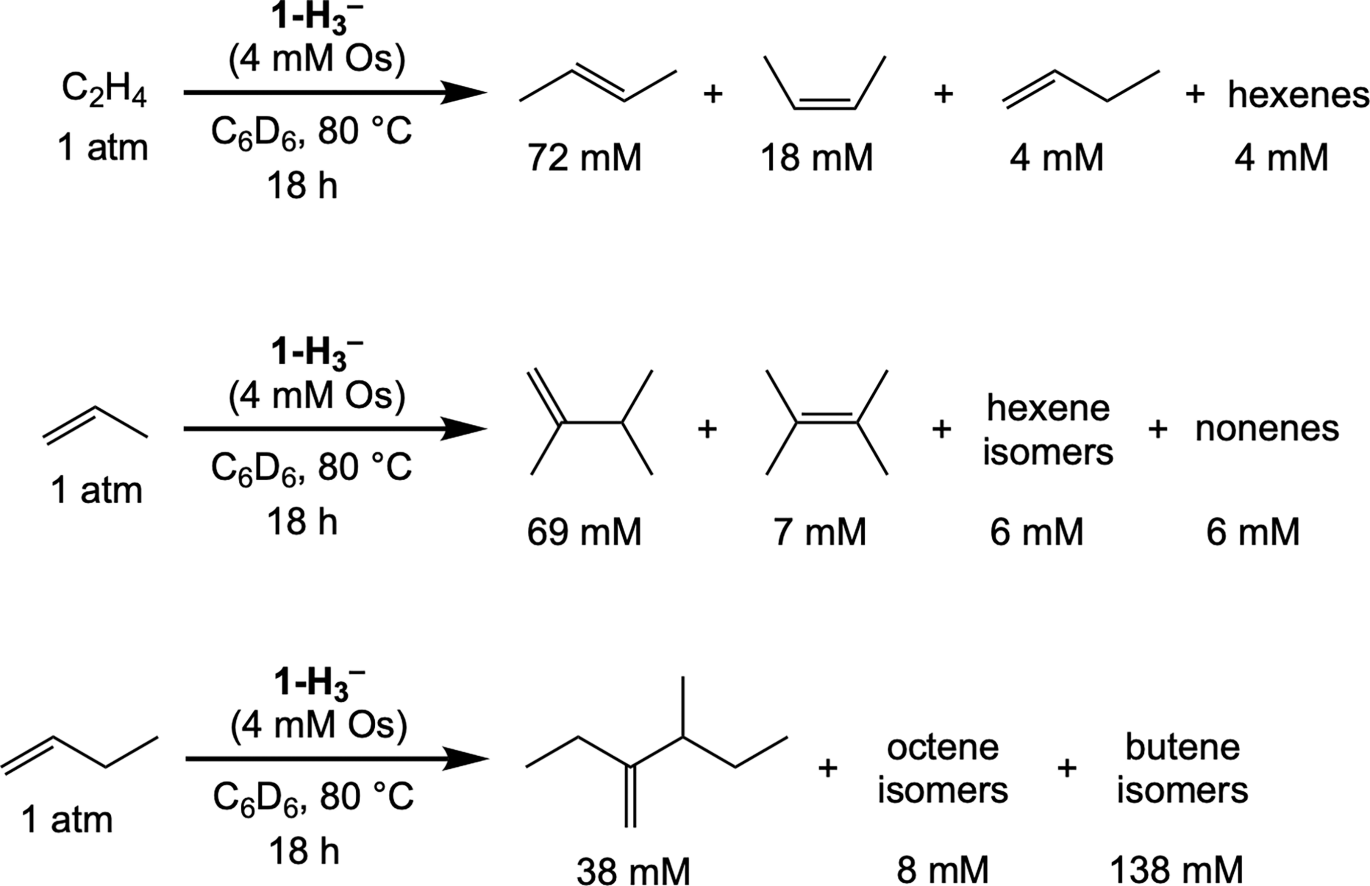
(Pybox)­Os-Catalyzed Olefin Homocoupling

Thus, in comparison with (Phebox)­Ir,^22^ the (Pybox)Os system was more reactive for olefin coupling (affording rates at 80 °C similar to those obtained at 110 °C with (Phebox)­Ir) and yielded butenes rather than butadiene.

We next investigated propene dimerization, a reaction that was not catalyzed to any significant extent by (Phebox)­Ir.^22^ Gratifyingly, the (Pybox)Os unit catalyzed propene dimerization almost as effectively as did ethylene dimerization. Only one major product was observed, 2,3-dimethylbut-1-ene, accompanied by much smaller amounts of the double-bond isomer 2,3-dimethylbut-2-ene, and other hexenes (Scheme 4). This is an unusual example of predominantly the *t-t* dimerization of propene. To our knowledge all examples of *t-t* dimerization of simple alkenes by molecular catalysts are believed to proceed through a metallacyclopentane mechanism,^18–21,31,32^ in accord with the observation of the osmacyclopentane here, as well as with the mechanism of olefin coupling previously proposed^22^ for (Phebox)­Ir.

1-Butene was also catalytically dimerized, analogously with propene dimerization, to give predominantly the *t-t* product, 3-methyl-4-methylenehexane (Scheme 4). The yields (ca. 40%) were somewhat lower than those obtained with propene, which we attribute to double-bond isomerization of the 1-butene and the apparent inability of the catalyst to couple internal olefins. With 1-hexene, only trace amounts of the dimerization product were observed. This likely reflects the smaller fraction of 1-hexene relative to 1-butene expected at equilibrium, given the accessibility of the three internal positions within the C_6_ chain.

No coupling was observed with 3,3-dimethylbut-1-ene (TBE), which can presumably be attributed to the steric bulk of the *t*-butyl groups. Styrene also gave no coupled product; this, however, appears to be a result of catalyst poisoning, as discussed below.

### Catalytic Olefin Cross-Coupling

Olefin mixtures were investigated with a view toward cross-coupling. Under an atmosphere of ethylene (0.6 atm) and propene (0.6 atm), a mixture of C_4_, C_5_, and C_6_ products was obtained from the homocoupling of ethylene, ethylene-propene cross-coupling, and propene homocoupling, respectively, yielding concentrations of 12, 27, and 5 mM, respectively. The major ethylene-propene cross-coupling products were 2-methyl-but-2-ene (19 mM) and its double-bond isomer, 2-methyl-but-1-ene (4 mM) (Scheme 5a). Qualitatively similar results were obtained with ethylene and 1-butene (Scheme 5b).

**Scheme 5:**
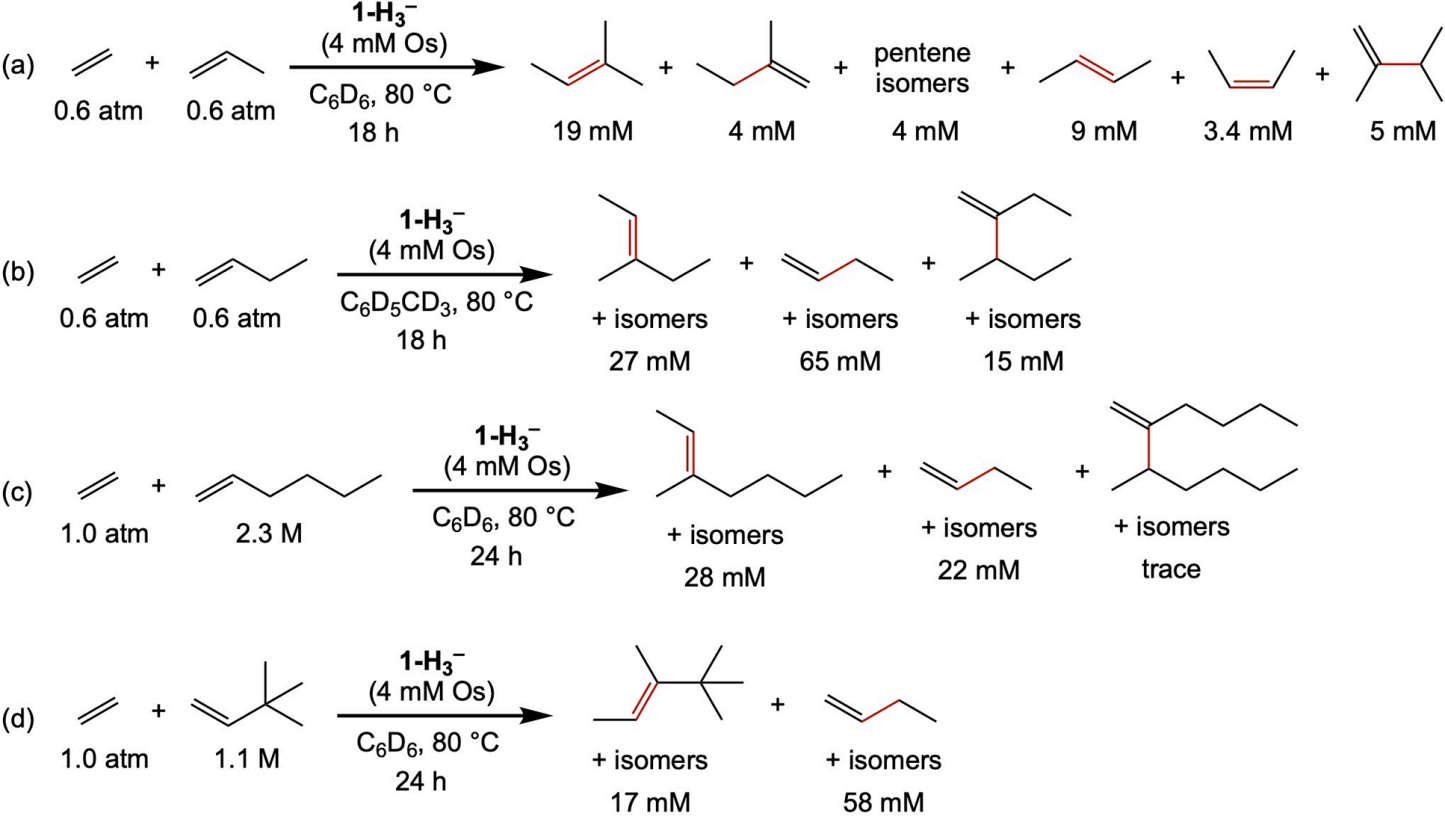
(Pybox)­Os-Catalyzed Olefin Cross-Coupling

1-Hexene, as noted above, gave little dimerization product in the absence of other olefins. A mixture of ethylene and 1-hexene, however, gave C_8_ cross-coupling products in yields comparable to ethylene homocoupling product (with only a trace of C_12_ product observed; Scheme 5c). This suggests that while 1-hexene homocoupling is slow relative to isomerization, ethylene-1-hexene cross-coupling is significantly faster. Likewise, while TBE did not undergo any significant homocoupling, either individually or in the presence of ethylene, it did undergo cross-coupling with ethylene to an appreciable extent (Scheme 5d).

In contrast, a mixture of styrene and ethylene gave no styrene dimer or cross-coupled product. But notably, ethylene homocoupling was also completely inhibited. Thus styrene is not merely unreactive; rather it acts as a catalyst poison. This likely results from chelation via addition of a terminal vinyl and ortho phenyl C–H bonds, to give a metalloindene, as we have previously described for (^iPr^PCP)Ir and various styrenes.^33^

## Discussion of the Mechanism and DFT calculations

A computational study of the mechanism of the (Pybox)­Os-catalyzed hydrovinylation, in conjunction with the mechanism of (Phebox)­Ir-catalyzed dehydrogenative coupling, elucidated surprising differences in the energetics of individual reaction steps, which explain the different levels of catalytic activity and, especially, the different chemoselectivity observed for the two systems. Geometry optimization and vibrational analysis were performed (gas phase) using the M06L density functional as incorporated in Gaussian 16 revA03.^34,35^ The basis set used for main group elements was 6–311G­(d,p),^36^ and for osmium and iridium, the relativistic effective core potential SDD was used with the associated basis set, along with an additional polarization function from the Frenking basis set.^37,38^ These calculations provided the correction terms to the Gibbs free energies at 298 K and 1 M.^39^ Additional single-point energies were computed in a polarizable continuum representing benzene as solvent^40^ using the M06, B3LYP-GD3BJ,^41,42^ ωb97XD,^43^ and PBE0-D3BJ^44,45^ density functionals. These calculations employed the def2tzvp basis set for the main group elements and def2qzvp for osmium and iridium and associated ECPs.^46,47^ No significant differences in the energy profiles are obtained with these variations. For the discussion in the text, we use the M06 results^48^ and we give the full results in the SI.

We have previously reported that elimination of HOAc from the reaction of (Phebox)­Ir­(OAc)H with NaO*^t^* Bu in the presence of ethylene resulted in the formation of the bis-ethylene complex (Phebox)­Ir­(C_2_H_4_)_2_ (**1** ^**Ir**^ **-(C**_**2**_ **H**_**4**_ **)**_**2**_).^49^ Heating a solution of **1** ^**Ir**^ **-(C**_**2**_ **H**_**4**_ **)**_**2**_ under an ethylene atmosphere at 100 °C afforded an equilibrium mixture of **1** ^**Ir**^ **-(C**_**2**_ **H**_**4**_ **)**_**2**_ and iridacyclopentane ethylene complex **2** ^**Ir**^ (ca. 30:70).^22^ In contrast, we never observe the analogous bis-ethylene osmium complex **1-(C**_**2**_ **H**_**4**_ **)**_**2**_ in the present system; instead, at room temperature, only the osmacyclopentane complex **2** is observed upon reaction with ethylene (Scheme 3). This is in accord with DFT calculations (Figure 3), which predict a barrier of only Δ*G* ^‡^ = 17.0 kcal/mol for the reaction of **1-(C**_**2**_ **H**_**4**_ **)**_**2**_ to yield **2** (via **TS1**, Figures 3 and 4), which then readily binds another equivalent of ethylene to give **2-C**_**2**_ **H**_**4**_.

**Figure 3:**
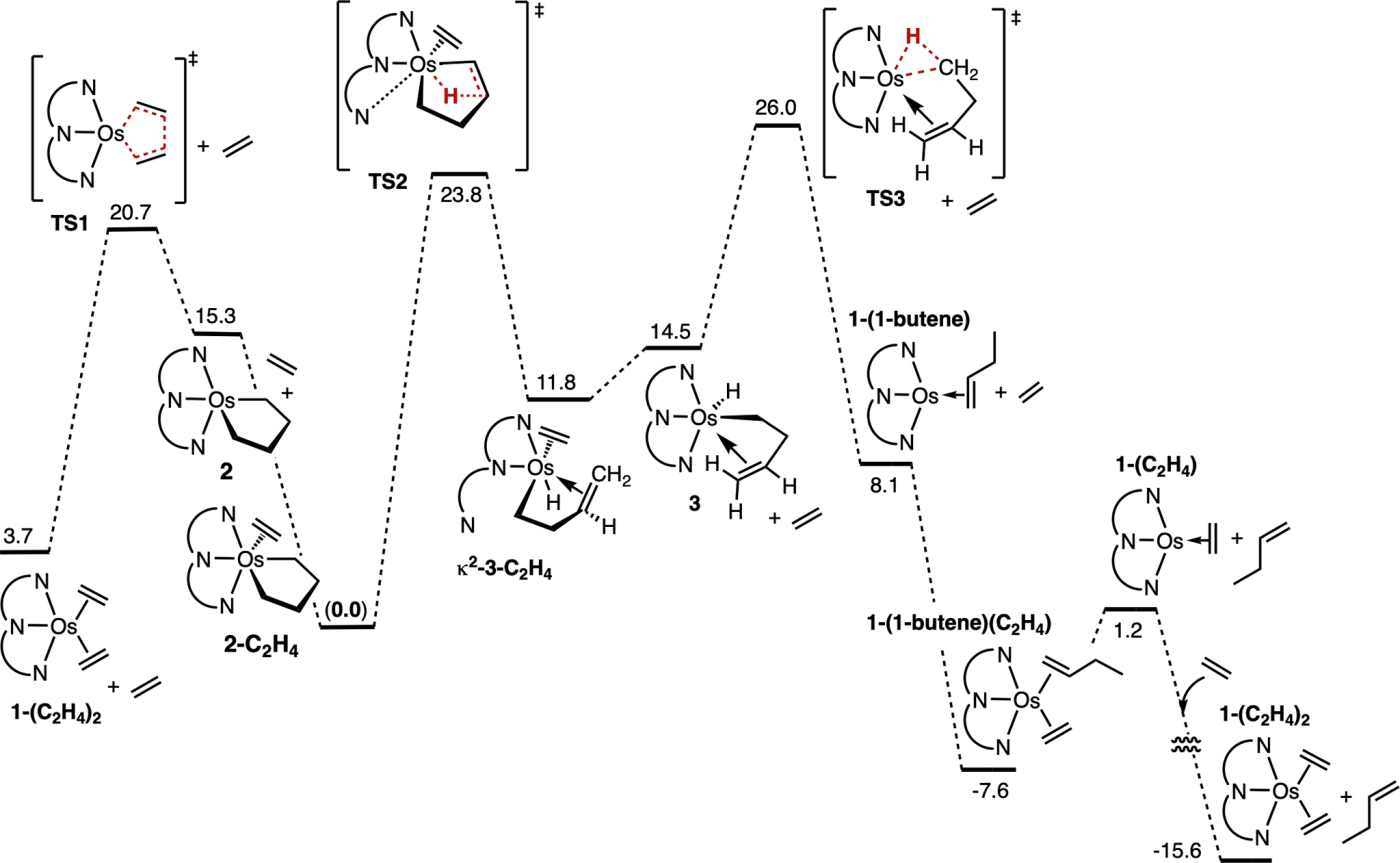
M06 Gibbs free energy profile for dimerization of ethylene catalyzed by **1** (in benzene solvent continuum at 298 K and 1 M). Values are normalized relative to **2-C**_**2**_ **H**_**4**_ and ethylene.

**Figure 4:**
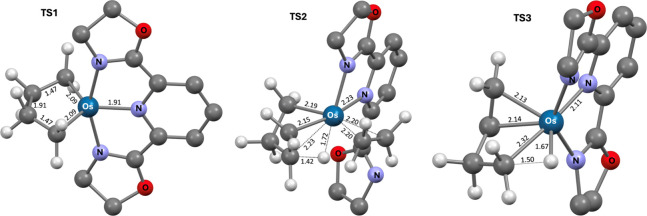
Calculated structures of key transition states (H atoms and methyl groups of Pybox ligand omitted for clarity; distances in Å).

**2-C**_**2**_ **H**_**4**_ is calculated to undergo κ^3^-κ^2^ dechelation of the Pybox ligand and β-H transfer, concertedly, to yield (κ^2^-Pybox)­Os­(κ^2^-3-butenyl)­(H)­(C_2_H_4_) (**κ** ^**2**^ **-3-C**_**2**_ **H**_**4**_) (via **TS2**, Figures 3 and 4). Loss of ethylene to give (κ^3^-Pybox)­Os­(κ^2^-3-butenyl)­(H), **3**, is then calculated to be only 2.7 kcal/mol endergonic. Reductive elimination of the hydride with the 3-butenyl σ-bond of **3** (via **TS3**, Figures 3 and 4) is then calculated to yield **1-(1-butene)**. A full cycle can then be completed via the addition of ethylene, loss of 1-butene, and addition of another ethylene molecule (Figure 3). The computations predict that the nominally highest barrier and therefore rate-determining step in the formation of 1-butene from ethylene is C–H reductive elimination from **3** (**TS3**); however, the transition state (**TS2**) calculated for β-H elimination and opening of the osmacyclopentane is, given the accuracy limits of the calculations, not unequivocally lower in energy than **TS3** (ΔG°**_TS3‑TS2_** = 2.2 kcal/mol).

Alternative pathways were computationally investigated. A TS for β-H elimination from complex **2** – *without* κ^3^-κ^2^ dechelation of the Pybox ligand or ethylene coordination as in **TS2** – was located, **TS2’** (Scheme 6). The free energy of this TS, however, was calculated to be very high, 17 kcal/mol above the β-H elimination transition state **TS2** shown in Figures 3 and 4. An alternative TS for C–H reductive elimination was located as well in which an additional molecule of ethylene is coordinated and the Pybox ligand is in the κ^2^ configuration, in contrast with the κ^3^-Pybox configuration of **TS3**. This transition state, **TS3′**, is calculated to be only 3.4 kcal/mol above the rate-determining **TS3**; considering the accuracy limits of the calculations, **TS3′** could be considered a viable transition state.

**Scheme 6:**
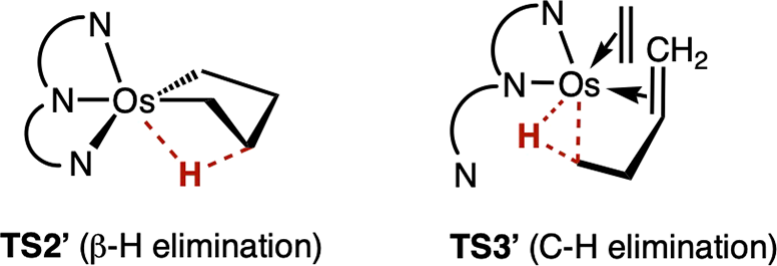
Alternative Transition States Calculated for β-H elimination and C–H Reductive Elimination

Kinetic investigation of ethylene dimerization sheds light on the question of the rate-limiting step in the cycle. The reaction rate was found to be first-order in the osmium catalyst, as expected for any mononuclear mechanism. More interestingly, when ethylene pressure was varied over a range from 1 to 5.4 atm, the rate was determined to be inverse first-order in ethylene pressure (Figure S30), indicative of a pre-equilibrium involving loss of ethylene followed by a rate-determining step. This result is consistent with the mechanism and energy profile indicated in Figure 3 (as expressed in eq 1), in which **TS3** is the rate-determining TS^50^ (RDTS), and it is inconsistent, in particular, with either **TS2** or **TS3′** being the RDTS.

$$2-C_{2}H_{4}\overset{K_{1}}{\underset{\left(\mathrm{via}\;\mathrm{TS}2\right)}{⇌}}3+C_{2}H_{4}\overset{K_{1}}{\underset{\left(\mathrm{via}\;\mathrm{TS}3\right)}{\to }}(\mathrm{Pybox})\mathrm{Os}(1-\mathrm{butene})+C_{2}H_{4}$$ 1

predicted: *k*_obs_ = *K*_1_ *k*_1_[**2-C**_**2**_ **H**_**4**_]­[C_2_H_4_]^−1^

### Comparison with (Phebox)­Ir

The mechanism of (Pybox)­Os-catalyzed ethylene dimerization is very closely related to the mechanism we proposed several years ago for dehydrogenative coupling of ethylene to butadiene catalyzed by the isoelectronic fragment (Phebox)­Ir.^22^ Initially, both mechanisms proceed via (pincer)­M­(ethylene)_2_, which undergoes cyclization and ethylene addition to give a metallacyclopentane ethylene complex.

As seen in Figure 5, the [2 + 2 + 1] cyclization reaction of the bis-ethylene complexes is somewhat less favorable, thermodynamically, for **1-(C**_**2**_ **H**_**4**_ **)**_**2**_ than for its Ir congener: Δ*G*° = 11.6 and 4.6 kcal/mol for (Pybox)Os and (Phebox)­Ir, respectively. The kinetic barriers, however, are strikingly lower for (Pybox)­Os than (Phebox)­Ir: Δ*G* ^‡^ = 17.0 and 25.9 kcal/mol, respectively, for the cyclization and 5.4 and 21.3 kcal/mol for the retrocyclization.

**Figure 5:**
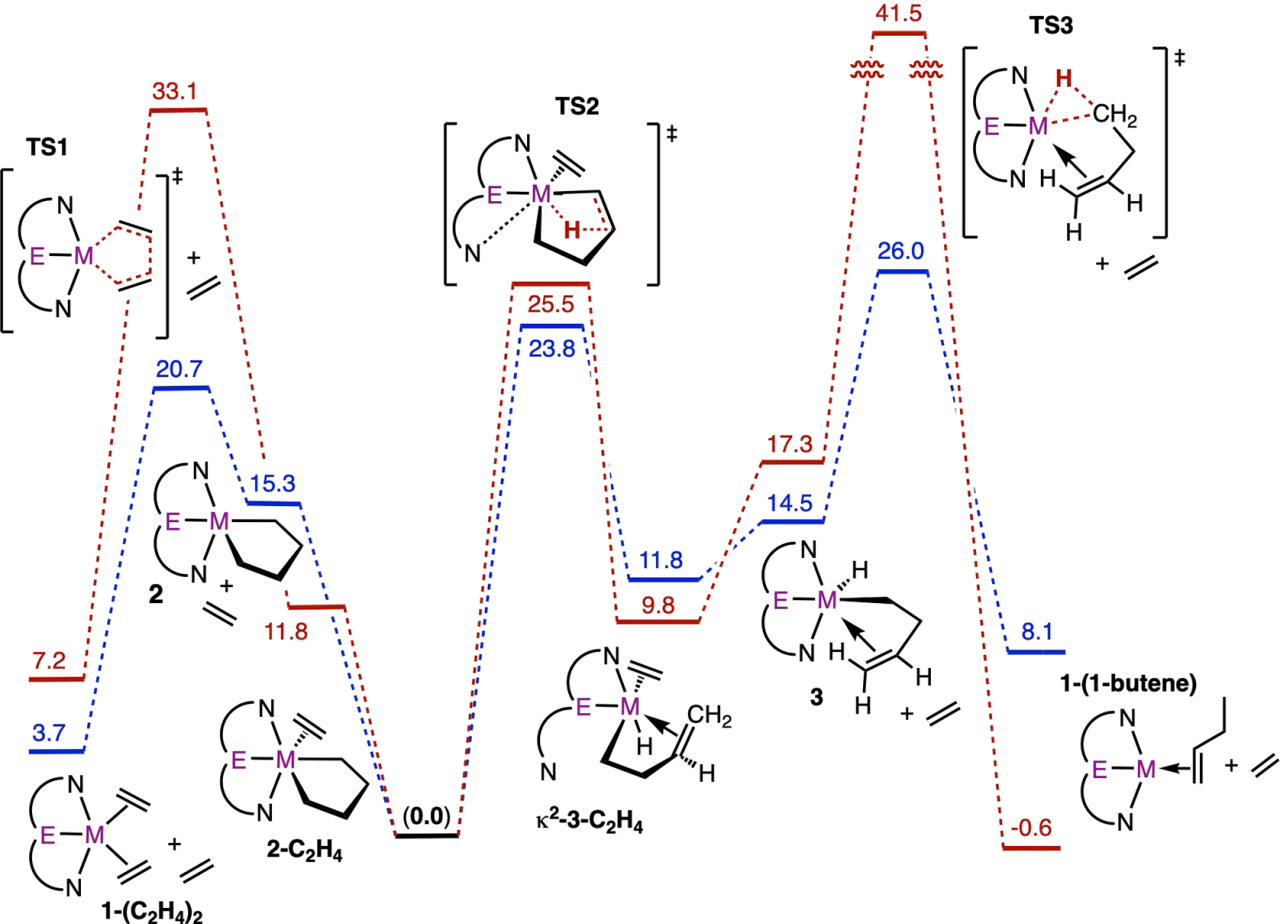
M06 Gibbs free energy profile for ethylene coupling by (Phebox)­Ir (red) and (Pybox)Os (blue) (in a benzene solvent continuum at 298 K and 1 M). Values are normalized relative to **2-C**_**2**_ **H**_**4**_. The mechanism of (Phebox)Ir is taken from ref 22, with free energies (kcal/mol) recalculated using the same method as used for (Pybox)Os in the present work.

The predicted RDTS for the pathways shown in Figure 5 for both (Pybox)­Os and (Phebox)­Ir, however, is not that for cyclization, **TS1**, but rather that for butenyl C–H reductive elimination, **TS3**. For (Phebox)­Ir, the barrier to butenyl C–H reductive elimination was found to be extremely high, 41.5 kcal/mol above that of **2** ^**Ir**^ **-C**_**2**_ **H**_**4**_. Accordingly 1-butene was not formed in the (Phebox)Ir systems. Instead, the butenyl unit dechelated to give a five-coordinate iridium σ-but-3-enyl hydride complex, which underwent β-H elimination (after insertion of ethylene into the Ir–H bond) to yield butadiene.^22^

In contrast with the prohibitively high overall barrier calculated for (Phebox)Ir (41.5 kcal/mol), the corresponding 1-butenyl C–H reductive elimination for (Pybox)Os was calculated to be accessible at 26.0 kcal/mol above **2-(C**_**2**_ **H**_**4**_ **)**. Within the accuracy limits of the calculations, this value is fully in agreement with the experimentally observed rates of (Pybox)­Os-catalyzed ethylene dimerization.

The large difference between the Ir and the Os systems in the overall barrier for the formation of 1-butene complexes (from **2** ^**Ir**^-**(C**_**2**_ **H**_**4**_ **)** and **2-(C**_**2**_ **H**_**4**_ **)**) is primarily attributable to the different kinetic barriers for the individual step of C–H reductive elimination from six-coordinate complexes **3** ^**Ir**^ and **3**. As might be expected, reductive elimination from Ir­(III) is more favorable than from Os­(II) (Δ*G*° = −17.9 kcal/mol versus −6.4 kcal/mol). Yet, remarkably, the kinetic barrier is much *greater* for elimination from Ir­(III) (Δ*G* ^‡^ = 24.2 versus 11.5 kcal/mol). This of course translates to an even much greater difference in the respective barriers to the reverse reaction, C–H addition: Δ*G* ^‡^ = 42.1 and 17.9 kcal/mol for the Ir and Os butene complexes respectively.

The but-3-enyl C–H reductive elimination TS is rate-determining in (Pybox)­Os-catalyzed coupling. The even higher barrier in the (Phebox)­Ir system precludes butene elimination, ultimately accounting for the formation of butadiene rather than butenes. In view of the importance of this step in the present systems – and the broader significance of C–H addition/elimination more generally^51^ – we extended our computational investigation of this reaction. In particular, we wished to determine if the relatively facile kinetics of the Os system were related to factors specific to the but-3-enyl ligand or if they are general for C–H reductive elimination from a (pincer)­M­(L)­(alkyl)­(H) complex where L is a π-coordinated olefin or perhaps even more broadly.

We first modeled the but-3-enyl C–H reductive elimination with C–H reductive elimination from methyl hydride complexes with a simple coordinated ethylene ligand (Scheme 7, where L = C_2_H_4_). Calculations established the same general difference between these (Phebox)Ir and (Pybox)Os methyl hydride complexes, as was found for the case of **3** and **3** ^**Ir**^. Specifically, computations predicted much more facile kinetics for C–H reductive elimination from the Os congener, yet also a much lower barrier to the reverse reaction, C–H addition to Os. We then examined C–H reductive elimination/oxidative addition of analogous complexes in which ethylene was replaced by iconic non-olefin ligands CO and PH_3_.

**Scheme 7:**
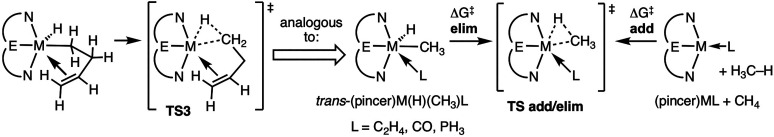
C–H Reductive Elimination from Butenyl Hydride and Methyl Hydride Complex

Table 1 shows the free energies of the C–H addition reactions, as well as the barriers to addition and elimination. As might be expected, C–H addition to the (Pybox)­Os^0^L complexes is thermodynamically much more favorable – by 13–17 kcal/mol – than addition to the corresponding (Phebox)­Ir^I^(L). Therefore, it is not surprising (it is consistent with the Hammond Postulate) that the barrier (Δ*G* ^‡^) to C–H addition to Os(0) is lower. Much more surprising, however, is the magnitude of the difference, ΔΔ*G* ^‡^: 21 to 28 kcal/mol, a difference greater than ΔΔ*G*°. It follows that the barrier to elimination from the six-coordinate Os methyl hydrides is *lower* than that from the Ir congeners (by 6–15 kcal/mol), despite elimination from Os being thermodynamically much *less* favorable. Table 1 also gives the average of the values of the forward and reverse barriers which may be considered as the intrinsic kinetic barrier (eq 2).^52,53^ This is seen to be 14–21 kcal/mol lower for the osmium than for the iridium complexes.

$$\mathrm{intrinsic}\;\mathrm{kinetic}\;\mathrm{barrier}=(\Delta {G^{‡}}_{\mathrm{add}}+\Delta {G^{‡}}_{\mathrm{elim}})/2=\Delta {G^{‡}}_{\mathrm{add}}-(\Delta {G_{\mathrm{add}}}^{{}^{\circ}}/2)$$ 2

**Table 1:** Activation and Reaction Free Energies of H_3_C–H Addition to (Pybox)­OsL and (Phebox)­IrL^a^

**M-L**	L	**M-L + CH**_**4**_	**Δ*G*** **_add_ **^‡^****	**Δ*G*_add**^°^**_**	**Δ*G*** **_elim_ **^‡^****	**int. kin. barr.** ^b^ **(Δ*G*** **_add_ **^‡^**** **+ Δ*G*** **_elim_ **^‡^**** **)/2**	**E-M-L (°)** ^c^	**Δ*G* bending E-M-L (120°)**
Ir(Phebox)	C_2_H_4_	0.0	35.8	19.1	16.7	**26.3**	176	16.7
Os(Pybox)		0.0	14.7	3.9	10.8	**12.8**	180	9.9
Ir(Phebox)	CO	0.0	46.6	25.6	21.0	**33.8**	179	16.4
Os(Pybox)		0.0	18.7	12.6^d^	6.1^d^	**12.4**	174	8.1
Ir(Phebox)	PH_3_	0.0	34.9	15.3	19.6	**27.3**	179	16.1
Os(Pybox)		0.0	11.3	–1.7	13.0	**12.2**	175	8.3
Ir(Phebox)	vacant	0.0	20.4	14.2	6.2	**13.3**	n.a.	n.a.
Os(Pybox)		0.0	7.8	–6.8	14.6	**11.2**	n.a.	n.a.

a Geometry optimization and vibrational analysis obtained (gas phase) using the M06 functional.

b Intrinsic kinetic barrier = (Δ*G*_add_^‡^ + Δ*G*_elim_^‡^)/2 = Δ*G*_add_^‡^ – (Δ*G*°_add_/2).

c E-M = C–Ir or N–Os; angle E-M-L where “L” is C of CO, P of PH_3_, and centroid of the ethylene C–C bond.

d The TS calculated for addition of H_3_C–H to (Pybox)­Os­(CO) also leads to an unusual *fac*-(Pybox)­Os­(H)­(CH_3_)­(CO) isomer with 7.7 kcal/mol lower Δ*G*° than the isomer considered herein (*mer, trans*-(Pybox)­Os­(H)­(CH_3_)­(CO); this would correspond to an intrinsic kinetic barrier for C–H addition/elimination of Δ*G* = 16.3 kcal/mol.

Similar to C–H addition to the four-coordinate Os and Ir complexes, addition to the three-coordinate (Pybox)­Os^0^ is 21 kcal/mol thermodynamically more favorable than to (Phebox)­Ir^I^. In contrast to the reactions of the four-coordinate complexes, however, these additions and eliminations follow Hammond-type behavior. Thus, in contrast to the four-coordinate complexes, ΔΔ*G* ^‡^ for C–H addition is less than ΔΔ*G*°, and the intrinsic kinetic barriers, (Δ*G*_add_^‡^ + Δ*G*_elim_^‡^)/2, are similar for the three-coordinate Os and Ir complexes (13.3 and 11.2 kcal/mol, respectively).

We note that the kinetic barriers to C–H reductive elimination from third-row (5d) d^6^ six-coordinate transition metal complexes, as well as the corresponding C–H additions, are commonly considered to be quite high, not only for Ir­(III) but also for Pt­(IV).^54^ This clearly has significant implications for catalysis by such species. The origin of the much lower intrinsic kinetic barriers for C–H addition/elimination of the four-coordinated Os complexes compared with the Ir congeners will, accordingly, be the subject of further study. For now, we tentatively propose that the underlying explanation relates to the point that the barrier to C–H addition to square planar d^8^ complexes is in large part a result of the need to bend one ligand out of the plane of the complex and the energetic cost thereof.^55,56^ Such bending from planarity is known to be much more favorable for complexes of Ru(0) and Os(0) than those of Rh­(I) and Ir­(I).^57–59^ In this context, we have calculated the energy of bending, to yield an E-M-L angle (E-M = C–Ir or N–Os) of 120° for the complexes investigated. The energetic penalty for both L = PH_3_ and L = CO is ca. 16 kcal/mol for all three Ir complexes and ca. 8 kcal/mol for the Os complexes (Table 1).

## Conclusions

(Pybox)Os precursors are found to be effective catalysts for the dimerization of ethylene and tail-to-tail (*t-t*) dimerization of propene. *t-t-*Dimerization of 1-butene is effected in moderate yield, while dimerization of higher 1-alkenes is more severely limited by double-bond isomerization to internal olefins, which are resistant to coupling. However, cross-coupling of ethylene with higher 1-alkenes (as well as propene) is moderately effective. Likewise, the sterically hindered olefin TBE does not undergo dimerization to any appreciable extent but can be cross-coupled with ethylene.

The mechanism has been investigated with particular focus on the comparison with the isoelectronic, isostructural (Phebox)Ir catalyst, which we have reported to effect dehydrogenative coupling of ethylene to yield butadiene. Like the (Phebox)Ir system, the (Pybox)Os cycle begins with a [2 + 2 + 1] cyclization of a bis-olefin complex to form a metallacyclopentane. Computations are in agreement with experimental observations, indicating that this cyclization has a much lower kinetic barrier for (Pybox)­Os­(ethylene)_2_. In both cases, this is followed by κ^3^-κ^2^ dechelation of the pincer ligand and β-H transfer to yield a σ–π-but-3-enyl hydride intermediate. The most important difference between the systems emerges subsequently: the Os complex undergoes C–H reductive elimination of the σ–π-coordinated butenyl ligand, which is the rate-determining step. The calculated free energy of the TS, 26 kcal/mol relative to the resting state, is consistent with the observed reaction rate, and the nature of the TS is consistent with an inverse dependence of the rate on P_C2H4_. In contrast, for (Phebox)­Ir, the overall barrier to this step is calculated to be prohibitive, 41 kcal/mol. Instead, as reported previously for the (Phebox)Ir catalyst, the butenyl π-bond decoordinates and (after insertion of another ethylene molecule into the Ir–H bond) the σ-but-3-enyl ligand undergoes β-H elimination to yield butadiene.

Thus, the key mechanistic distinction is the much more facile C­(sp^3^)-H elimination from the Os σ–π-but-3-enyl hydride (ΔG^‡^ = 11.5 kcal/mol vs 24.2 kcal/mol for the Ir congener). This is particularly striking since the much more facile elimination from Os has a much lower driving force (Δ*G*° = −6.4 kcal/mol vs −17.9 kcal/mol for Ir), corresponding to an extremely large difference in the barriers to C–H addition (Δ*G* ^‡^ = 17.9 kcal/mol vs 42.1 kcal/mol for Os and Ir systems, respectively). In view of the importance of C–H reductive elimination from six-coordinate d^6^ systems and the microscopic reverse,^51,54,56^ we have computationally investigated the generality of this phenomenon. Computations predict that the intrinsic kinetic barrier to C–H addition/elimination for the *trans*-(pincer)­MH­(L)­(CH_3_)/(pincer)­ML couple) is generally far greater for (Phebox)Ir than for (Pybox)Os – but no such difference is predicted for addition to the corresponding three-coordinate (pincer)­M species or for the microscopic reverse. Elucidation of the origin of this effect will be the subject of further study.

## Supplementary Material





## Notes

Complete experimental details and synthetic procedures, NMR data, computational data, computed energies and thermodynamic quantities (PDF)
Optimized structures for calculated species (.mol format) (ZIP)

## References

[1] Parshall, G. W. ; Ittel, S. D. Homogeneous Catalysis: The Applications and Chemistry of Catalysis by Soluble Transition Metal Complexes, 2 *nd ed.* Wiley: Hoboken, NJ, 1992.

[2] Chauvin, Y. ; Olivier, H. , Dimerization and Codimerization. In Applied Homogeneous Catalysis with Organometallic Compounds, Eds.; Cornils, B. ; Herrmann, W. A. , Eds. VCH: New York, 1996; Vol. 1, pp 258–268.

[3] Nomura K., Ishino M., Hazama M., Suzukamo G. (1997). Efficient
synthesis
of 2,3-dimethylbutenes by dimerization of propylene using nickel-phosphine
catalyst in the presence of strong sulfonic acids and/or dialkyl sulfates.
Remarkable effect of strong sulfonic acids and/or dialkyl sulfates. J. Mol. Catal. A: Chem..

[4] Svejda S. A., Brookhart M. (1999). Ethylene Oligomerization and Propylene Dimerization
Using Cationic (a-Diimine)­nickel­(II) Catalysts. Organometallics.

[5] Marchionna M., Di Girolamo M., Patrini R. (2001). Light olefins dimerization to high
quality gasoline components *Catal*. Today.

[6] Olivier-Bourbigou, H. ; Saussine, L. , Dimerization and codimerization. In Applied Homogeneous Catalysis with Organometallic Compounds *(* 2 *nd Edition)*, 2002; Vol. 1, pp 253–265.

[7] Suzuki Y., Yasumoto T., Mashima K., Okuda J. (2006). Hafnocene Catalysts
for Selective Propylene Oligomerization: Efficient Synthesis of 4-Methyl-1-pentene
by β-Methyl Transfer. J. Am. Chem. Soc..

[8] Lang J. R. V., Denner C. E., Alt H. G. (2010). Homogeneous
catalytic dimerization
of propylene with bis­(imino)­pyridine vanadium­(III) complexes. J. Mol. Catal. A.

[9] Deimund M. A., Labinger J., Davis M. E. (2014). Nickel-Exchanged
Zincosilicate Catalysts
for the Oligomerization of Propylene. ACS Catal..

[10] Sarazen M. L., Doskocil E., Iglesia E. (2016). Effects of Void Environment
and Acid Strength
on Alkene Oligomerization Selectivity ACS Catal..

[11] Comito R. J., Metzger E. D., Wu Z., Zhang G., Hendon C. H., Miller J. T., Dincă M. (2017). Selective
Dimerization of Propylene
with Ni-MFU-4l. Organometallics.

[12] Olivier-Bourbigou H., Breuil P. A. R., Magna L., Michel T., Espada Pastor M. F., Delcroix D. (2020). Nickel Catalyzed Olefin
Oligomerization and Dimerization. Chem. Rev..

[13] Ling Y., Chen X., Tong H., Guan W., Chen P., Huang Z., Liang C. (2021). Modulating
the Interaction of NiSO4
and Nb2O5 Boosts the Dimerization of Propylene. Ind. Eng. Chem. Res..

[14] Petit J., Magna L., Mézailles N. (2022). Alkene oligomerization
via metallacycles:
Recent advances and mechanistic insights *Coord*. Chem. Rev..

[15] Luo Z., Gao J., Li M., Wen Z., Li H. (2024). Bis­(imino)­pyridine
vanadium (III) and cobalt (II) complexes catalyzed selective dimerization
of propylene *Molecular*. Catalysis.

[16] Alzamly A., Bakiro M., Hussein
Ahmed S., Siddig L. A., Nguyen H. L. (2022). Linear
α-olefin oligomerization and polymerization catalyzed by metal-organic
frameworks *Coord*. Chem. Rev..

[17] Nomura K., Ishino M., Suzukamo G. (1997). Synthesis
of 2,3-Dimethylbutenes
by Dimerization of Propene Using Highly Active Nickel-Phosphine Catalysts
in the Presence of Sulfonic Acids and/or Dialkyl Sulfates *Bull*. Chem. Soc. Jpn..

[18] Ho C.-Y., He L. (2010). Catalytic Intermolecular
Tail-to-Tail Hydroalkenylation of Styrenes
with α Olefins: Regioselective Migratory Insertion Controlled
by a Nickel/N-Heterocyclic Carbene *Angew*. Chem., Int. Ed..

[19] Russell S. K., Lobkovsky E., Chirik P. J. (2011). Iron-Catalyzed Intermolecular [2π + 2π]
Cycloaddition. J. Am. Chem. Soc..

[20] McLain S. J., Schrock R. R. (1978). Selective olefin dimerization via tantallocyclopentane
complexes. J. Am. Chem. Soc..

[21] Schrock R., McLain S., Sancho J. (1980). Tantalacyclopentane
complexes and
their role in the catalytic dimerization of olefins *Pure and
Appl*. Chem..

[22] Gao Y., Emge T. J., Krogh-Jespersen K., Goldman A. S. (2018). Selective Dehydrogenative
Coupling of Ethylene to Butadiene via an Iridacyclopentane Complex. J. Am. Chem. Soc..

[23] Parihar, A. ; Malakar, S. ; Chakraborty, S. ; Gallo, M. C. ; Emge, T. J. ; Hasanayn, F. ; Goldman, A. S. Olefin Coupling Catalyzed by (Pybox)Os Complexes via Osmacyclopentane Intermediates. Comparison with Isoelectronic (Phebox)­Ir. ChemRxiv 2025, DOI: 10.26434/chemrxiv-2025-wvffr. PMC12728604 41450661

[24] Nishiyama H., Sakaguchi H., Nakamura T., Horihata M., Kondo M., Itoh K. (1989). Chiral and C2-symmetrical bis­(oxazolinylpyridine)­rhodium­(III) complexes:
effective catalysts for asymmetric hydrosilylation of ketones. Organometallics.

[25] Luo S.-X., Tiwow V., Maeder M., Lawrance G. A. (2010). Synthesis and metal­(II)
ion complexation of pyridine-2,6-diamides incorporating amino alcohols. J. Coord. Chem..

[26] Lu D.-F., Zhu C.-L., Jia Z.-X., Xu H. (2014). Iron­(II)-Catalyzed
Intermolecular Amino-Oxygenation of Olefins through the N–O
Bond Cleavage of Functionalized Hydroxylamines. J. Am. Chem. Soc..

[27] Lease N., Pelczar E. M., Zhou T., Malakar S., Emge T. J., Hasanayn F., Krogh-Jespersen K., Goldman A. S. (2018). PNP-Pincer Complexes
of Osmium: Comparison with Isoelectronic (PCP)Ir and (PNP)­Ir^+^ Units. Organometallics.

[28] Crabtree, R. H. The Organometallic Chemistry of the Transition Metals 6th ed.; John Wiley & Sons: Hoboken, NJ, 2014; p 85.

[29] Fantoni T., Palladino C., Grigolato R., Muzzi B., Ferrazzano L., Tolomelli A., Cabri W. (2025). Mastering palladium-catalyzed cross-coupling
reactions: the critical role of in situ pre-catalyst reduction design *Org*. Chem. Front..

[30] Consistent with the role of a proton source in this reaction, treatment of a THF solution of **1-H** _ **3** _ ^ **–** ^ with 3 equiv [H(OEt_2_)_2_][BArF^24^] led to the appearance of multiple upfield (δ < 0) resonances in the ^1^H NMR spectrum, indicative of the formation of several hydride-containing species. Upon treatment of this mixture with 1 atm of ethylene, the complex **2-C** _ **2** _ **H** _ **4** _ was generated w*ithin 10 min at room temperature* (vs ca. 12 h in the absence of added acid)

[31] Ting C., Messerle L. (1987). Intermolecular vinylic
carbon-hydrogen bond activation
by a doubly-bonded organoditantalum complex. J. Am. Chem. Soc..

[32] For an example of propene dimerization to 2,3-dimethylbutene catalyzed by a nickel-based system that may be molecular, but is not well characterized, see references 3 and 17.

[33] Wilklow-Marnell M., Li B., Zhou T., Krogh-Jespersen K., Brennessel W. W., Emge T. J., Goldman A. S., Jones W. D. (2017). Catalytic Dehydrogenative
C–C Coupling by a Pincer-Ligated Iridium Complex. J. Am. Chem. Soc..

[34] Zhao Y., Truhlar D. G. (2006). A new local density functional for main-group thermochemistry,
transition metal bonding, thermochemical kinetics, and noncovalent
interactions. J. Chem. Phys..

[35] Frisch, M. J. ; Trucks, G. W. ; Schlegel, H. B. ; Scuseria, G. E. ; Robb, M. A. ; Cheeseman, J. R. ; Scalmani, G. ; Barone, V. ; Petersson, G. A. ; Nakatsuji, H. ; Li, X. ; Caricato, M. ; Marenich, A. V. ; Bloino, J. ; Janesko, B. G. ; Gomperts, R. ; Mennucci, B. ; Hratchian, H. P. ; Ortiz, J. V. ; Izmaylov, A. F. ; Sonnenberg, J. L. ; Williams-Young, D. ; Ding, F. ; Lipparini, F. ; Egidi, F. ; Goings, J. ; Peng, B. ; Petrone, A. ; Henderson, T. ; Ranasinghe, D. ; Zakrzewski, V. G. ; Gao, J. ; Rega, N. ; Zheng, G. ; Liang, W. ; Hada, M. ; Ehara, M. ; Toyota, K. ; Fukuda, R. ; Hasegawa, J. ; Ishida, M. ; Nakajima, T. ; Honda, Y. ; Kitao, O. ; Nakai, H. ; Vreven, T. ; Throssell, K. ; Montgomery, J. A., Jr. ; Peralta, J. E. ; Ogliaro, F. ; Bearpark, M. J. ; Heyd, J. J. ; Brothers, E. N. ; Kudin, K. N. ; Staroverov, V. N. ; Keith, T. A. ; Kobayashi, R. ; Normand, J. ; Raghavachari, K. ; Rendell, A. P. ; Burant, J. C. ; Iyengar, S. S. ; Tomasi, J. ; Cossi, M. ; Millam, J. M. ; Klene, M. ; Adamo, C. ; Cammi, R. ; Ochterski, J. W. ; Martin, R. L. ; Morokuma, K. ; Farkas, O. ; Foresman, J. B. ; Fox, D. J. Gaussian 16, Revision D.01, Gaussian, Inc.: Wallingford, CT, 2016.

[36] Krishnan R., Binkley J. S., Seeger R., Pople J. A. (1980). Self-consistent
molecular orbital methods. XX. A basis set for correlated wave functions. J. Chem. Phys..

[37] Andrae D., Haeussermann U., Dolg M., Stoll H., Preuss H. (1990). Energy-adjusted
ab initio pseudopotentials for the second and third row transition
elements *Theor*. Chim. Acta.

[38] Ehlers A. W., Böhme M., Dapprich S., Gobbi A., Höllwarth A., Jonas V., Köhler K. F., Stegmann R., Veldkamp A., Frenking G. (1993). A set of f-polarization functions for pseudo-potential
basis sets of the transition metals Sc–Cu, Y–Ag and
La–Au *Chem*. Phys. Lett..

[39] Cramer, C. J. Essentials of Computational Chemistry: Theories and Models, 2 *nd ed.* Wiley: 2004.

[40] Marenich A. V., Cramer C. J., Truhlar D. G. (2009). Universal
Solvation Model Based on
Solute Electron Density and on a Continuum Model of the Solvent Defined
by the Bulk Dielectric Constant and Atomic Surface Tensions. J. Phys. Chem. B.

[41] Lee C., Yang W., Parr R. G. (1988). Development of the Colle-Salvetti
correlation-energy formula into a functional of the electron density *Phys*. Rev. B.

[42] Grimme S., Ehrlich S., Goerigk L. (2011). Effect of
the damping function in
dispersion corrected density functional theory. J. Comput. Chem..

[43] Chai J.-D., Head-Gordon M. (2008). Long-range corrected hybrid density functionals with
damped atom–atom dispersion corrections *Phys*. Chem. Chem. Phys..

[44] Antony J., Sure R., Grimme S. (2015). Using dispersion-corrected
density
functional theory to understand supramolecular binding thermodynamics *Chem*. Commun..

[45] Adamo C., Barone V. (1999). Toward reliable density
functional methods without
adjustable parameters: The PBE0 model. J. Chem.
Phys..

[46] Weigend F., Ahlrichs R. (2005). Balanced basis sets
of split valence, triple zeta valence
and quadruple zeta valence quality for H to Rn: Design and assessment
of accuracy *Phys*. Chem. Chem.
Phys..

[47] Weigend F. (2006). Accurate Coulomb-fitting
basis sets for H to Rn *Phys*. Chem. Chem. Phys..

[48] Das S., Mandal S., Malakar S., Emge T. J., Goldman A. S. (2024). Bis­(N-xylyl-imino)­phenyl
″NCN″ iridium pincer complexes. Thermodynamics of ligand binding and C-C bond cleavage Polyhedron.

[49] Gao Y., Guan C., Zhou M., Kumar A., Emge T. J., Wright A. M., Goldberg K. I., Krogh-Jespersen K., Goldman A. S. (2017). β-Hydride Elimination and C–H
Activation
by an Iridium Acetate Complex, Catalyzed by Lewis Acids. Alkane Dehydrogenation
Cocatalyzed by Lewis Acids and [2,6-Bis­(4,4-dimethyloxazolinyl)-3,5-dimethylphenyl]­iridium. J. Am. Chem. Soc..

[50] Kozuch S., Shaik S. (2011). How to Conceptualize Catalytic Cycles? The Energetic Span Model *Acc*. Chem. Res..

[51] c Goldberg, K. I. ; Goldman, A. S. , Activation and Functionalization of C—H Bonds; American Chemical Society: 2004; Vol. 885. 10.1021/bk-2004-0885.

[52] Yang J.-D., Chen B.-L., Zhu X.-Q. (2018). New Insight
into the Mechanism of
NADH Model Oxidation by Metal Ions in Nonalkaline Media. J. Phys. Chem. B.

[53] Endicott J. F., Balakrishnan K. P., Wong C.-L. (1980). Oxidation-reduction
reactions of
complexes with macrocyclic ligands. Energetics and dynamics of methyl
exchange. J. Am. Chem. Soc..

[54] Wick D. D., Goldberg K. I. (1997). C-H Activation at
Pt­(II) To Form Stable Pt­(IV) Alkyl Hydrides. J. Am. Chem. Soc..

[55] Dedieu A., Strich A. (1979). A molecular orbital analysis of the oxidative addition
of hydrogen to the chlorotris­(triphenylphosphine)­rhodium­(I) complex *Inorg*. Chem..

[56] Saillard J., Hoffmann R. (1984). C-H and H-H Activation
in Transition Metal Complexes
and on Surfaces. J. Am. Chem. Soc..

[57] Ogasawara M., Macgregor S. A., Streib W. E., Folting K., Eisenstein O., Caulton K. G. (1995). Isolable, Unsaturated Ru(0) in Ru­(CO)_2_(P*
^t^
*Bu_2_Me)_2_: Not Isostructural
with Rh­(I). J. Am. Chem. Soc..

[58] Ogasawara M., Macgregor S. A., Streib W. E., Folting K., Eisenstein O., Caulton K. G. (1996). Characterization and Reactivity of an Unprecedented
Unsaturated Zero-Valent Ruthenium Species: Isolable, Yet Highly Reactive. J. Am. Chem. Soc..

[59] Das S., Pal A. K., Datta A. (2024). Pressure Induced Alteration and Stabilization
of Intermolecular Stacking in Square-planar Osmium tetracarbonyl. ChemPhysChem.

