# Supplementary material for: Olefin Coupling Catalyzed by (Pybox)Os Complexes via Osmacyclopentane Intermediates: Comparison with Isoelectronic (Phebox)Ir
Source: JACS Au. 2025 Dec 9;5(12):6220–30. doi: 10.1021/jacsau.5c01197 (PMC12728604; doi:10.1021/jacsau.5c01197)
Supplement: Supplementary file 1 [file au5c01197_si_001.pdf]

Supporting Information for:

Olefin Coupling Catalyzed by (Pybox)Os Complexes via Osmacyclopentane Intermediates. Comparison with Isoelectronic (Phebox)Ir

Ashish Parihar[a], Santanu Malakar[b], Soham Chakraborty[a], Michael C. Gallo[a], Thomas J. Emge[a], Faraj Hasanayn[c]*, Alan S. Goldman[a]*

[a]*Department of Chemistry and Chemical Biology, Rutgers, The State University of New Jersey, New Brunswick, New Jersey 08903, United States*

[b]*Department of Chemistry, Rutgers University–Camden, Camden, New Jersey 08102, United States*

[c]*Department of Chemistry, American University of Beirut, Beirut 1107 2020, Lebanon*

*Email: fh19@aub.edu.lb; alan.goldman@rutgers.edu

Table of Contents

S1. General considerations **S2**
a) General procedure for homocoupling of olefins **S3**
b) General Procedure for cross-coupling of olefins **S3**

S2. Synthesis
a) (^{dm}Pybox)-ligand **S3**
b) (^{dm}Pybox)$OsCl_3$ (**1-Cl$_3$**) **S4**
c) [(^{dm}Pybox)OsH_3][K(THF)$_2$] ([**1-H$_3$**]K(THF)$_2$ **S5**
d) [(^{dm}Pybox)OsH_3][Na(THF)$_2$] ([**1-H$_3$**]Na(THF)$_2$) **S5**
e) (^{dm}Pybox)Os(cy-C_4H_8)(C_2H_4) (**2-C$_2$H$_4$**) **S6**
f) (^{dm}Pybox)Os(cy-C_4H_8)(PMe_3) (**2-PMe$_3$**) **S7**

S3. NMR Spectra **S8**

S4. Crystallographic Data
a) ^{dm}Pybox **S21**
b) (^{dm}Pybox)$OsCl_3$ (**1-Cl$_3$**) **S22**
c) [(^{dm}Pybox)OsH_3][K(THF)$_2$] ([**1-H$_3$**]K(THF)$_2$ **S23**

S5. Kinetic Studies **S25**

S6. Computational Data **S27**

S7. References **S30**

S1. General Considerations

All reactions were conducted under argon atmosphere in an MBraun glovebox or using a Schlenk line unless otherwise specified. All glassware was cleaned thoroughly and dried in an oven maintained at 140 °C for at least 8 hours. Anhydrous THF, dichloromethane, benzene, toluene, and pentane were all obtained from Sigma-Aldrich and stored over molecular sieves in a Strauss flask, under an argon atmosphere and used without further purification. Deuterated solvents were obtained from Cambridge Isotope Labs and were degassed with three freeze-pump-thaw cycles on the Schlenk line and then dried by stirring over neutral alumina overnight before being stored over molecular sieves inside the glovebox for use. NMR spectra were acquired on 500-MHz Varian and 500-MHz Bruker NMR spectrometers. ^{1}H and ^{13}C NMR spectra have been referenced to residual solvent peaks. ^{31}P NMR spectra were referenced to a PMe_3 standard. All scXRD data were collected on a Rigaku XTALab Synergy-S Single crystal diffractometer. Gas Chromatographic analyses (FID detection) were performed on a Varian 430-GC instrument equipped with an Agilent J&W GS-GasPro column (60 m length x 0.32 mm ID) using the following method:

FID starting temperature: 40 °C
Time at starting temp: 1.4 min
Ramp 1: 20 °C/min up to 200 °C with hold time = 3 min Ramp 2: 30 °C/min up to 260 °C with hold time = 70.6 min
Flow rate (carrier): 1.4 mL/min (N_2)
Split ratio: 25
Inlet temperature: 250 °C
Detector temperature: 260 °C.

S1.a. General procedure for homocoupling of olefins.

In an argon-filled glovebox, a J-Young NMR tube was charged with catalyst (2.0 μmol) followed by addition of 0.5 mL benzene-d_6 or toluene-d_8. Mesitylene was used as the standard for quantification. In the case of liquid olefins, the desired substrate was added in the glovebox and the NMR tube was then removed from the glovebox and heated at 80 °C for 18 h, the reaction was monitored by GC, and the final data was used to calculate the TON of the reaction using the formula:

$$\mathrm{TON} = \frac{[\text{mesitylene}] \times (\text{coupled product}_{\text{integral}}) \times (\text{no. C atoms in mesitylene (9)})}{(\text{mesitylene}_{\text{integral}}) \times (\text{no. of C atoms in coupled product}) \times [\text{catalyst}]}$$

For gaseous olefins, a J-Young NMR tube was charged with catalyst (2.0 μmol) followed by addition of 0.5 mL of benzene-d_6 or toluene-d_8, and then mesitylene standard. The J-Young NMR tube was removed from the glovebox, the argon atmosphere was evacuated on the Schlenk line, and the desired gaseous olefin was then added (1-2 atm). The J-Young NMR tube was then heated at 80 °C in an oven equipped to enable rotation of the tube to effect gas-liquid mixing. The final data point was determined by GC, as well as NMR spectroscopy (in some cases H/D exchange hindered quantification by NMR spectroscopy), with comparison to product standards.

S1.b. General Procedure for cross-coupling of olefins.

In an argon-filled glovebox, a J-Young NMR tube was charged with catalyst (2.0 μmol) followed by addition of 0.5 mL of benzene-d_6 or toluene-d_8, and then mesitylene standard. In the case of liquid olefins as coupling partners, the desired substrate was added in the glovebox. The argon atmosphere was then removed on the Schlenk line and the desired gaseous substrate olefin was added (0.6-1 atm). The J-Young NMR tube was then heated at 80 °C in an oven equipped for rotation to enable gas/liquid mixing. The final data for the reaction after 18 h was quantified by GC.

For the heterocoupling of two gaseous olefins, a J-Young NMR tube was charged with catalyst (2.0 μmol) followed by addition of 0.5 mL of benzene-d_6 or toluene-d_8. The mesitylene standard was then added to the J-Young NMR tube which was removed from the glovebox, the argon atmosphere was removed on the Schlenk line, and the desired gaseous substrate olefin was then added (0.6 atm). Without evacuating the headspace of the J-Young NMR tube, it was charged with the second olefin (0.6 atm) and then heated in an oven at 80 °C equipped for rotation. The final data was obtained by GC and NMR spectroscopy (in some cases H/D exchange hindered the quantification by NMR spectroscopy), with comparison to product standards.

S2. Synthesis

S2.a. Synthesis of ^{dm}Pybox ligand.

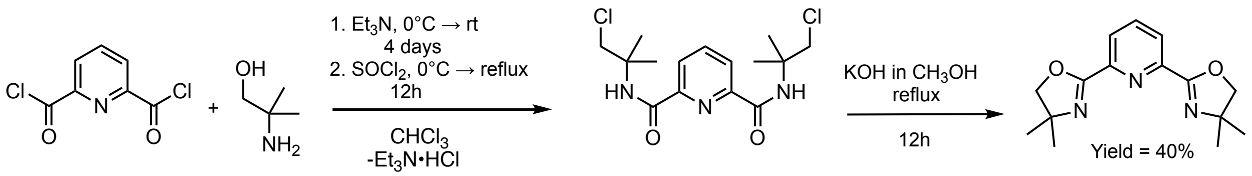

Scheme S1. Synthesis of 2,6-bis(4,4-dimethyl-4,5-dihydrooxazol-2-yl) pyridine (^{dm}Pybox)

2,6-bis(4,4-dimethyl-4,5-dihydrooxazol-2-yl)pyridine (^{dm}Pybox or "Pybox") was synthesized according to a modified literature procedure[S1, 2]. In a Schlenk flask, 2,6-pyridinedicarbonyl dichloride (2.31 g, 11.3 mmol) was dissolved in anhydrous $CHCl_3$. 2-amino-2-methylpropan-1-ol (2.02 g, 22.6 mmol) and triethylamine (4.72 mL, 33.9 mmol) were dissolved in $CHCl_3$ in a different Schlenk flask, and the solution was brought to 0 °C. The 2,6-pyridinedicarbonyl dichloride solution was then added slowly and dropwise to the second solution. The reaction mixture was brought to room temperature generating a pink colored solution which was then stirred for 4 days in argon atmosphere at room temperature.

After 4 days, without any workup, the mixture was cooled to 0 °C, and 10 equiv of $SOCl_2$ (8.2 mL, 0.113 mol) was added very slowly dropwise to the reaction mixture. The pink solution becomes almost colorless. The mixture was then refluxed for 12 h at 65 °C. Workup of the reaction involved evaporating solvent and excess $SOCl_2$ and quenching the reaction mixture initially with saturated $NaHCO_3$ solution and then with water. The product was extracted with ethyl acetate and dried with Na_2SO_4, and removal of solvent in vacuo afforded yellowish liquid N^2,N^6-bis(1-chloro-2-methylpropan-2-yl)pyridine-2,6-dicarboxamide as the product. (Yield 3.64 g.)

To effect cyclization, N^2,N^6-bis(1-chloro-2-methylpropan-2-yl)pyridine-2,6-dicarboxamide (3.64 g, 10.5 mmol) was dissolved in anhydrous methanol which was cooled to 0 °C. A methanol solution of KOH (5.91 g, 105 mmol) was added slowly to this solution, and the reaction was slowly brought to room temperature and then refluxed at 65 °C overnight. The methanol solvent was then evaporated, and the yellow solid cake was extracted with hexanes. The removal of hexane solvent in vacuo afforded the white solid as the product 2,6-bis(4,4-dimethyl-4,5-dihydrooxazol-2-yl) pyridine (Pybox). Yield = 1.26 g (40 %). X-ray quality crystals were obtained by slow evaporation of a pentane/hexane solution of Pybox ligand in air, affording a molecular structure with one molecule H_2O hydrogen bonded to both N-atoms of Pybox. (**Figure S26**)

^{1}H NMR (500 MHz, benzene-*d*$_6$) δ 8.06 (d, *J* = 7.8 Hz, 2H), 6.91 (t, *J* = 7.9 Hz, 1H), 3.74 (s, 4H), 1.13 (s, 12H).

^{13}C NMR (126 MHz, benzene-*d*$_6$) δ 161.68, 148.05, 136.46, 125.68, 79.39, 68.02, 28.39.

S2.b. Synthesis of (Pybox)OsCl$_3$ (1-Cl$_3$).

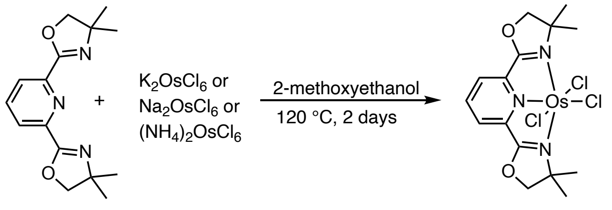

Scheme S2. Synthesis of (^{dm}Pybox)OsCl$_3$ (1-Cl$_3$).

In an argon-filled glovebox, a Schlenk flask was charged with ^{dm}Pybox (200 mg, 0.732 mmol), followed by addition of K_2OsCl_6 (352 mg, 0.732 mmol)[S3]. The flask was removed from the glovebox and 20 mL of 2-methoxyethanol was added on the Schlenk line. The reaction mixture was heated under argon atmosphere for two days at 120 °C, leading to the formation of a purple-red solution. The 2-methoxyethanol solvent was then removed in vacuo, followed by the addition of 8-10 mL of dichloromethane. The solution was then subjected to canula filtration, and the filtrate was cooled down to -40 °C before adding ether (30 mL). The solution was stirred at -40 °C for 10-15 min leading to the precipitation of purple-red solid from the solution, which was then filtered by canula filtration; the solid residue was further washed with ether (10 mL) at -40° C. The solid generated was dried further on the Schlenk line, generating pure (^{dm}Pybox)OsCl$_3$ (**1-Cl$_3$**). Yield, 260 mg (62 %). The **1-Cl$_3$** is stable under air for prolonged times without any decomposition. The ^{1}H NMR spectrum of paramagnetic **1-Cl$_3$** ($CDCl_3$) displayed signals in the far upfield and downfield regions (**Figure S3**). Crystals suitable for X-ray diffraction were obtained by slow evaporation of diethyl ether into a dichloromethane solution of **1-Cl$_3$** at room temperature. The molecular structure obtained was approximately octahedral (see details in crystallographic section **S4b**).

^{1}H NMR (500 MHz, Chloroform-*d*) δ 14.22 (methylene CH_2 s, 4H), 2.44 (CH_3 s, 12H), -3.21 (para-H s, 1H), -33.33 (meta-H s, 2H).

Elemental Analysis: Anal. Calcd for (^{dm}Pybox)OsCl$_3$ C, 31.61; H, 3.36; N, 7.37; Found: C, 32.08; H, 3.52; N, 7.15.

S2.c. Synthesis of [K(THF)$_2$][(^{dm}Pybox)OsH$_3$] ([K(THF)$_2$][1-H$_3$]).

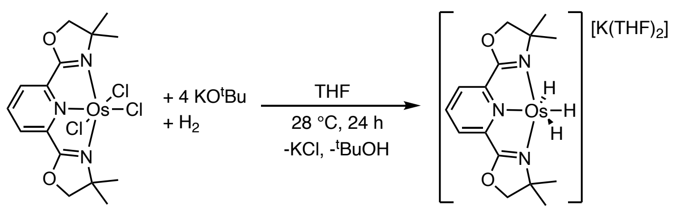

In an argon-filled glovebox, a 25-mL Schlenk flask was charged with (^{dm}Pybox)OsCl$_3$ (**1-Cl$_3$**) (100 mg, 0.174 mmol), followed by addition of THF solvent (15 mL) affording a purple-red solution. The flask was removed from the glovebox and charged with 1 atm of H_2. 4 equiv KO^{t}Bu (78 mg, 0.696 mmol) was then added under a continuous flow of argon. The reaction was then stirred at room temperature under H_2 atmosphere for 24 h, generating a blackish brown solution. The excess H_2 was removed from the Schlenk flask, and the solution was filtered via canula filtration. The solvent was then removed in vacuo and the solid was washed with pentane. Yield: 60 mg (54 %). Crystals for X-ray structure determination were obtained by vapor diffusion of pentane into a THF solution at room temperature. The formation of [**1-H$_3$**][K(THF)$_2$], which includes an upfield (-13.18 ppm) singlet (3H) in the ^{1}H NMR spectrum (THF-d$_8$) corresponding to metal hydride signals, along with signals attributable to the ligand backbone. The ^{1}H-^{1}H COSY (**Figure S5**) shows a strong correlation between ligand backbone protons.

^{1}H NMR (500 MHz, THF-*d*$_8$) δ 7.66 (d, *J* = 7.4 Hz, 2H), 6.38 (t, *J* = 7.4 Hz, 1H), 4.57 (s, 4H), 1.50 (s, 12H), -13.18 (s, 3H).
^{13}C NMR (126 MHz, THF-*d*$_8$) δ 157.69, 127.54, 110.13, 105.51, 80.63, 69.80, 25.86.

S2.d. Synthesis of [(^{dm}Pybox)OsH$_3$][Na(THF)$_2$] ([1-H$_3$]Na(THF)$_2$.

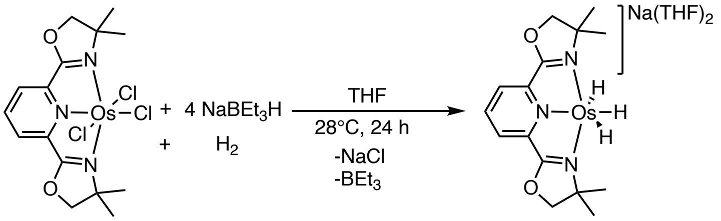

In an argon-filled glovebox, a 50-mL Schlenk flask was charged with (^{dm}Pybox)OsCl$_3$ (**1-Cl$_3$**) (100 mg, 0.174 mmol), followed by the addition of THF solvent (15 mL) forming a purple-red solution. The flask was removed from the glovebox and charged with 1 atm of H_2. 4 equiv of NaBEt$_3$H (0.75 mL, 0.696 mmol) was then added under a continuous flow of argon. The Schlenk flask was then re-charged with H_2, following which the reaction was stirred at room temperature under H_2 atmosphere for 24 h generating blackish-brown solution. The H_2 atmosphere was evacuated from the Schlenk flask, and the solution was filtered via canula filtration. Pentane (40 mL) was added to the filtrate at -40 °C, resulting in precipitation of the product. Canula filtration was then performed again and the solid was dried in vacuo. The solid was washed again with pentane (10 mL). Yield 70 mg (63%). The formation of [**1-H$_3$**][Na(THF)$_2$] was evident from the ^{1}H NMR spectrum (THF-d$_8$), which includes an upfield (-14.0 ppm) singlet (3H)

corresponding to metal hydride signals, along with signals attributable to the ligand backbone. The ^{1}H NMR and ^{1}H-^{1}H COSY attributable to the ligand backbone signals is essentially the same as found for [**1-H$_3$**][K(THF)$_2$].

^{1}H NMR (500 MHz, THF-*d*$_8$) δ 7.54 (d, *J* = 7.5 Hz, 2H), 6.43 (t, *J* = 7.5 Hz, 1H), 4.58 (s, 4H), 1.47 (s, 12H), -14.04 (s, 3H).

S2.e. Reaction of [(^{dm}Pybox)OsH$_3$]M ([1-H$_3$]M(THF)$_2$ with ethylene to generate 2-C$_2$H$_4$.

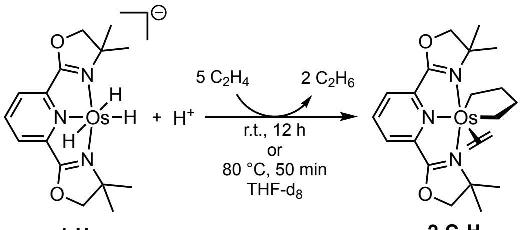

1-H$_3^-$ **2-C$_2$H$_4$**

In an argon-filled glovebox, a J-Young NMR tube was charged with [M][**1-H$_3^-$**] (both Na$^+$ and K$^+$ salts are effective; see NMR Spectra section S7-S14) (10 mg), followed by addition of THF-d$_8$ (0.5 mL), generating a blackish brown colored solution. The tube was removed from the glovebox, and 1 atm of ethylene was charged on the Schlenk line to the reaction mixture at room temperature. The tube was further kept at room temperature for 12 h with rotation to ensure gas/solution mixing, resulting in a color change from blackish brown to light brown. In an alternative procedure, the tube was heated for 50 min at 80 °C in an oven equipped with rotation to achieve solution/gas mixing. The characterization of the product by NMR spectroscopy in both the cases indicated the formation of **2-C$_2$H$_4$**. When ethylene was removed from solution, **2-C$_2$H$_4$** decomposes to several unidentified products as revealed by NMR. Addition of CO[S4] also led to several unidentified products, one of which is **2-(CO)$_2$**. Thus all attempts to obtain isolated product led to decomposition and the product was characterized in situ by ^{1}H NMR, ^{1}H-^{1}H COSY, ^{1}H-^{13}C gHSQC, and ^{13}C NMR spectroscopy (Figure S7-S14).

^{1}H NMR (500 MHz, THF-*d*$_8$) δ 7.27 (d, *J* = 7.6 Hz, 2H), 6.93 (t, *J* = 7.6 Hz, 1H), 4.53 (d, *J* = 8.1 Hz, 2H), 4.47 (t, *J* = 6.8 Hz, 2H), 4.38 (d, *J* = 8.2 Hz, 2H), 2.48 (s, 4H), 2.16 (p, *J* = 6.8 Hz, 2H), 1.64 (p, *J* = 7.1 Hz, 1H), 1.57 (s, 12H), 0.88 – 0.86 (m, 2H).

^{13}C NMR (126 MHz, THF-*d*$_8$) δ 173.13, 142.98, 127.65, 115.23, 83.55, 74.51, 51.60, 42.91, 36.97, 27.76, 27.31, 24.55, 12.39.

S2.f. Reaction of 2-C$_2$H$_4$ with PMe$_3$ yielding (^{dm}Pybox)Os(cy-C$_4$H$_8$)(PMe$_3$) (2-PMe$_3$).

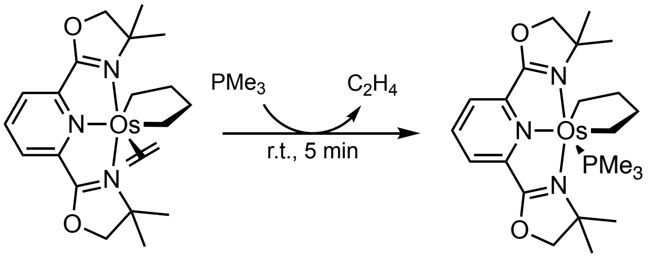

As noted in section **S2.e**, we were unable to isolate solid **2-C_2H_4**. In an effort to obtain a different, hopefully isolable osmacyclopentane complex, **2-C_2H_4** was generated in situ and treated with excess PMe_3 (6 equiv), under ethylene atmosphere, resulting in a color change from light brown to dark green within 5 minutes. ^{1}H NMR spectroscopy revealed the loss of bound ethylene signals at 2.48 ppm and a signal was found in the ^{31}P{^{1}H} NMR spectrum at -72.78 ppm. Evaporation of solvent and scratching the solid from the J-Young NMR tube resulted in the formation of **2-PMe_3** in 90-95 % purity. The characterization of the compound by ^{1}H NMR spectroscopy indicated a shift of the signals due to the C1 and C4 protons of the osmacyclopentane from 4.47 ppm and 0.88 ppm for **2-C_2H_4,** to 6.87 ppm and -0.47 ppm in **2-PMe_3** (**Figures S7, S10** and **S15**). The signals for the C1 and C4 protons of the appear as quartets for **2-PMe_3** rather than triplets in the case of **2-C_2H_4**, due to ^{31}P-^{1}H coupling; with ^{31}P decoupling the ^{1}H{^{31}P} NMR spectrum showed the same multiplicity pattern as **2-C_2H_4** (**Figure S17**). The product was further characterized by ^{1}H-^{1}H COSY, ^{1}H-^{13}C gHSQC, and ^{13}C NMR spectroscopy. (See NMR Spectra Section).

^{1}H NMR (500 MHz, THF-*d*$_8$) δ 7.05 (d, *J* = 7.6 Hz, 2H), 6.77 (q, *J* = 6.9 Hz, 2H), 6.53 (t, *J* = 7.7 Hz, 1H), 4.71 (d, *J* = 8.0 Hz, 2H), 4.38 (d, *J* = 8.1 Hz, 2H), 2.37 (p, *J* = 6.9 Hz, 2H), 1.69 (s, 6H), 1.57 (s, 6H), 1.41 – 1.33 (m, 2H), 0.54 (d, *J* = 7.3 Hz, 9H), -0.46 (q, *J* = 7.2 Hz, 2H).

^{31}P NMR (202 MHz, THF-*d*$_8$) δ -72.78.

^{13}C NMR (126 MHz, THF-*d*$_8$) δ 173.43 (d, *J* = 2.2 Hz), 145.70, 122.69, 114.54, 82.99, 75.45, 46.04 (d, *J* = 18.6 Hz), 35.41, 28.37, 26.40, 20.97 (d, *J* = 58.0 Hz), 14.17 (d, *J* = 22.1 Hz), 0.90 (d, *J* = 7.3 Hz).

S3. NMR Spectra

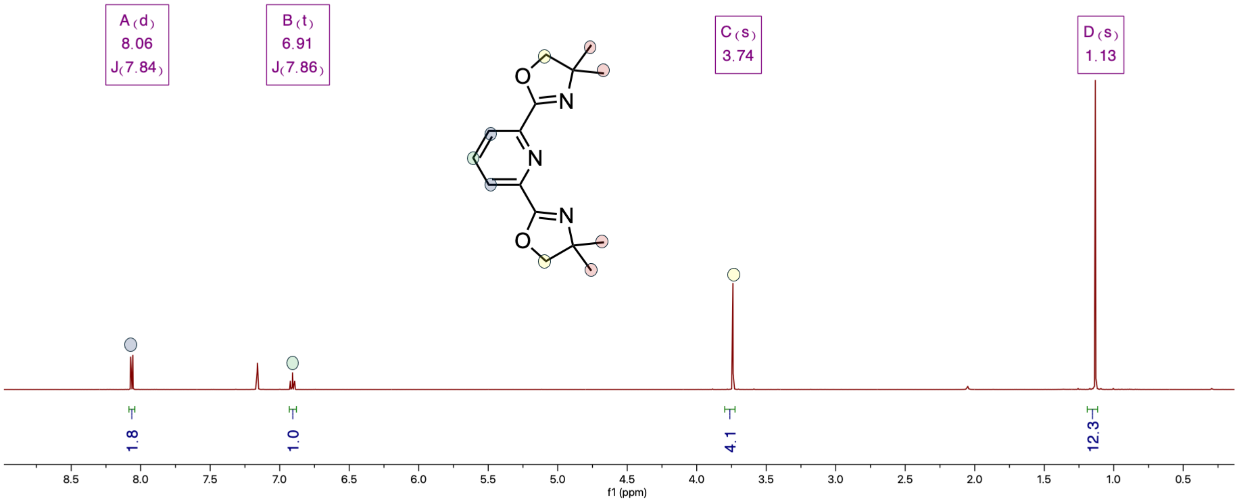

Figure S1: ^{1}H NMR spectrum of ^{dm}Pybox in benzene-d_6 at 298 K.

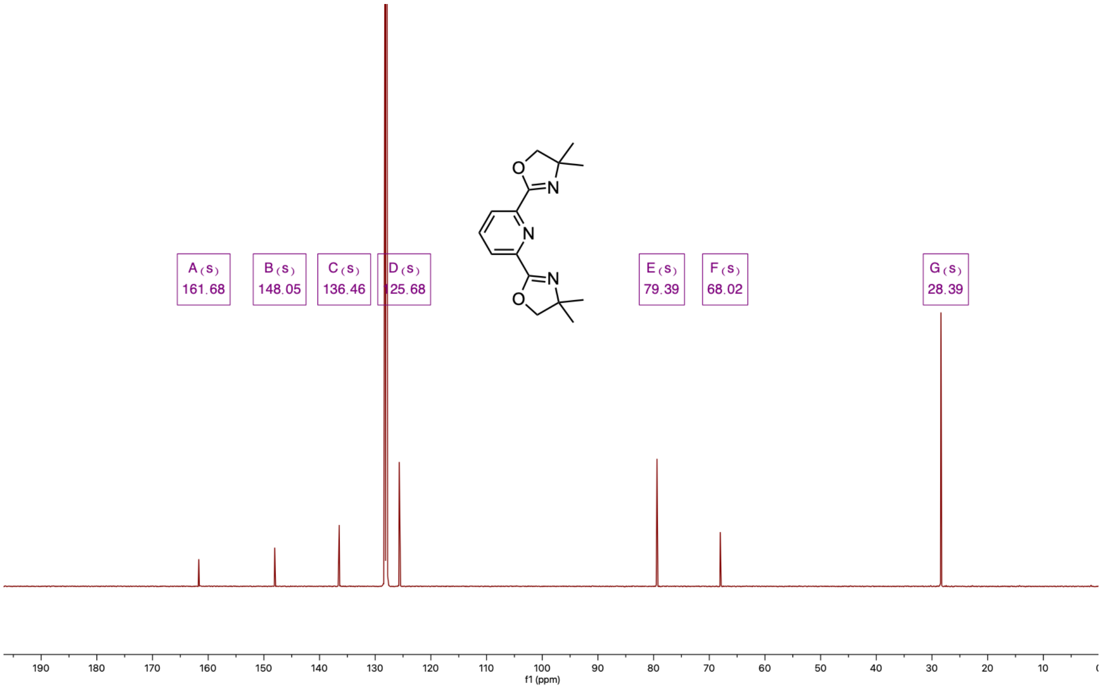

Figure S2. ^{13}C NMR spectrum of ^{dm}Pybox in benzene-d_6 at 298 K.

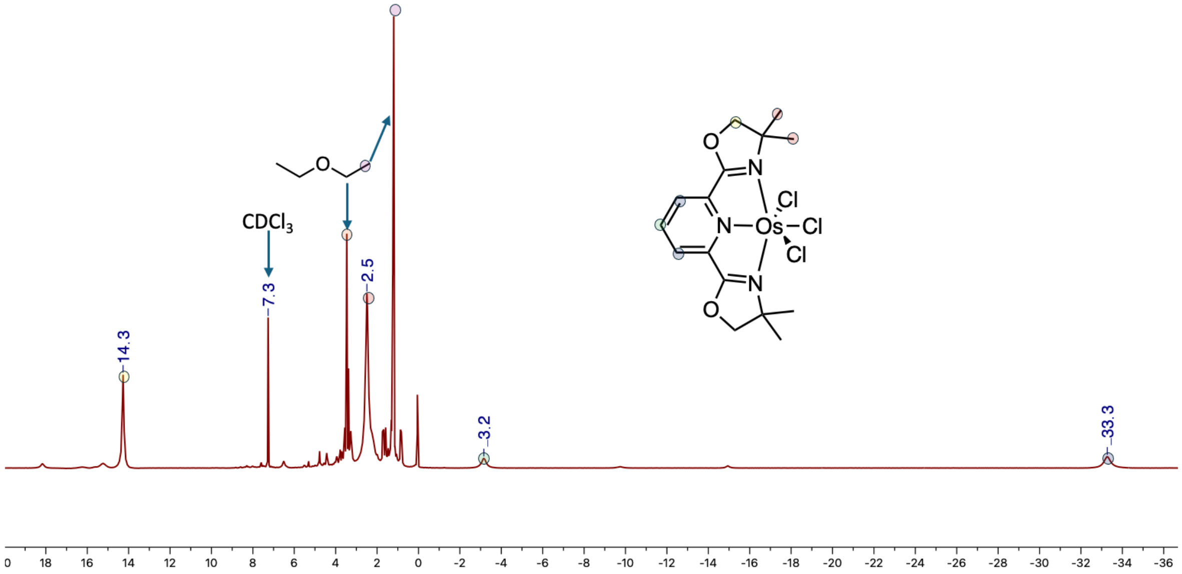

Figure S3. ^{1}H NMR spectrum of (^{dm}Pybox)$OsCl_3$ (**1-Cl$_3$**) in $CDCl_3$ at 298 K.

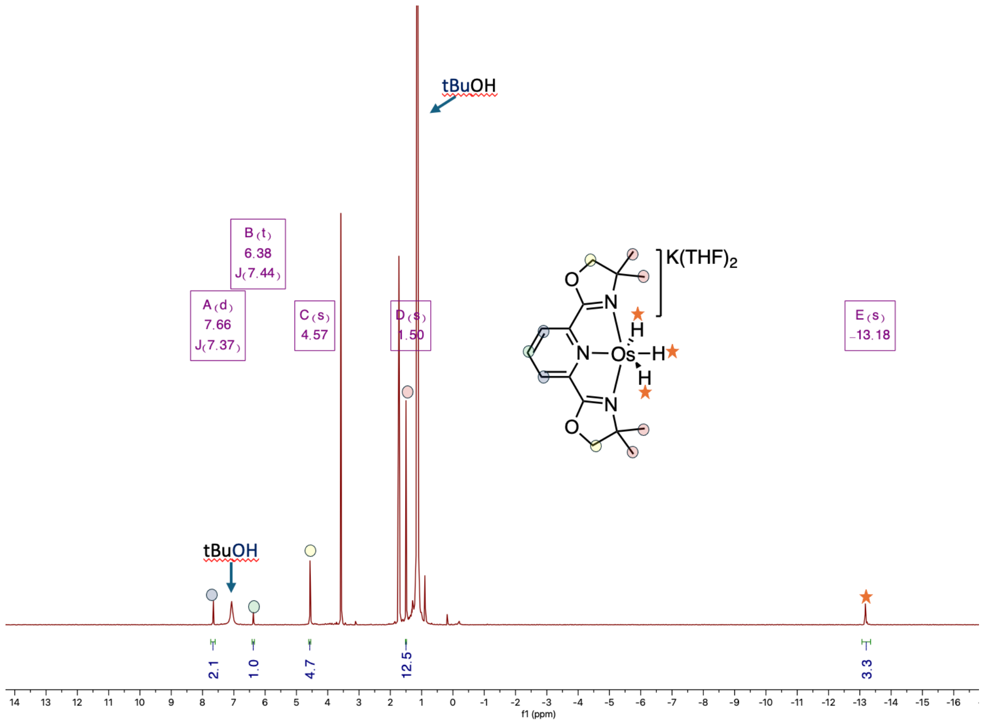

Figure S4. ^{1}H NMR spectrum of [(^{dm}Pybox)OsH_3]K ([**1-H$_3$]K**) in THF-d_8 at 298 K.

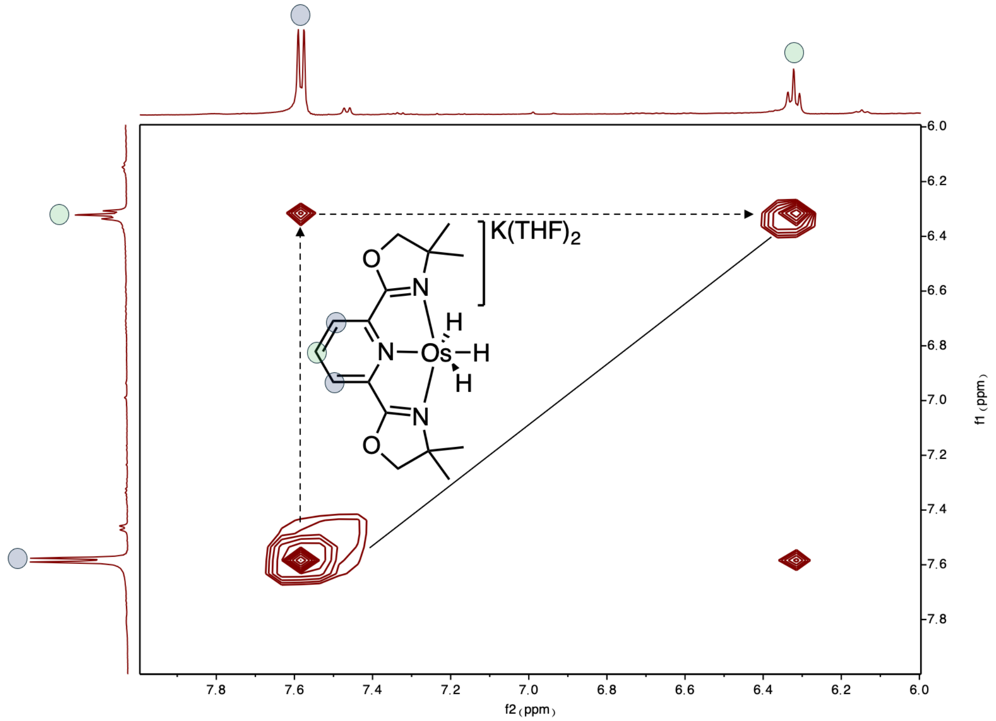

Figure S5. ^{1}H-^{1}H COSY NMR spectrum of [(^{dm}Pybox)OsH_3]K (**[1-H$_3$]K**) in THF-d_8 at 298 K.

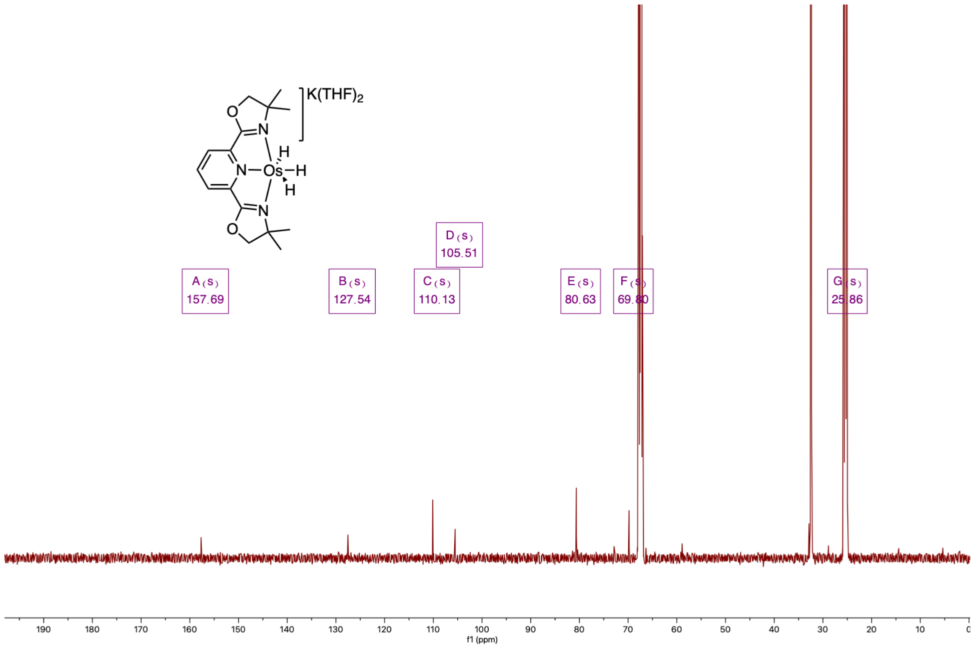

Figure S6. ^{13}C NMR spectrum of [(^{dm}Pybox)OsH_3]K (**[1-H$_3$]K**) in THF-d_8 at 298 K.

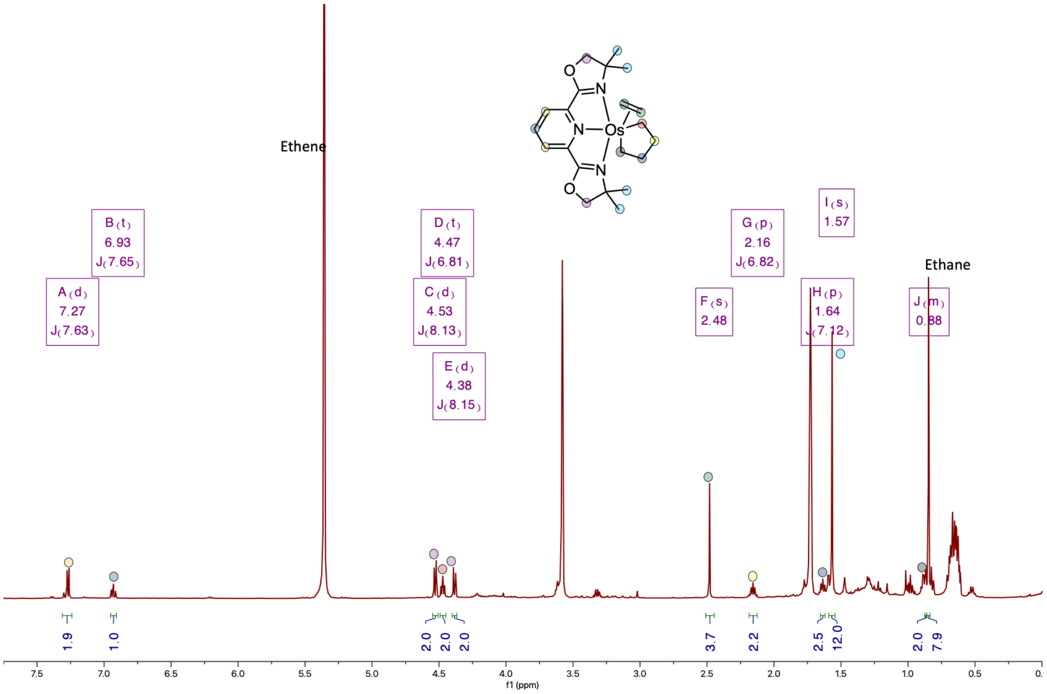

Figure S7. ^{1}H NMR spectrum of reaction of $[(^{dm}Pybox)OsH_3]Na$ ($[\mathbf{1\text{-}H_3}]Na$) with ethylene generating $\mathbf{2\text{-}C_2H_4}$ $[(^{dm}Pybox)Os(cy\text{-}C_4H_8)(C_2H_4)$ in THF-d_8 at 298 K

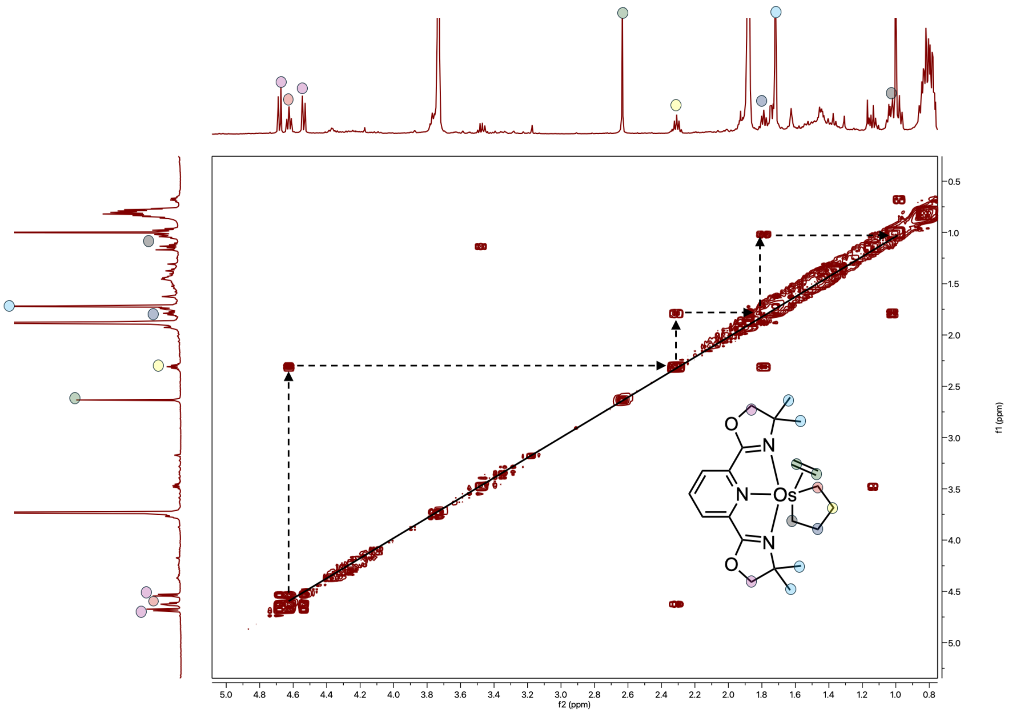

Figure S8. ^{1}H-^{1}H COSY NMR spectrum of reaction of $[(^{dm}Pybox)OsH_3]Na$ ($[\mathbf{1\text{-}H_3}]Na$) with ethylene generating $\mathbf{2\text{-}C_2H_4}$ $[(^{dm}Pybox)Os(cy\text{-}C_4H_8)(C_2H_4)$ in THF-d_8 at 298 K.

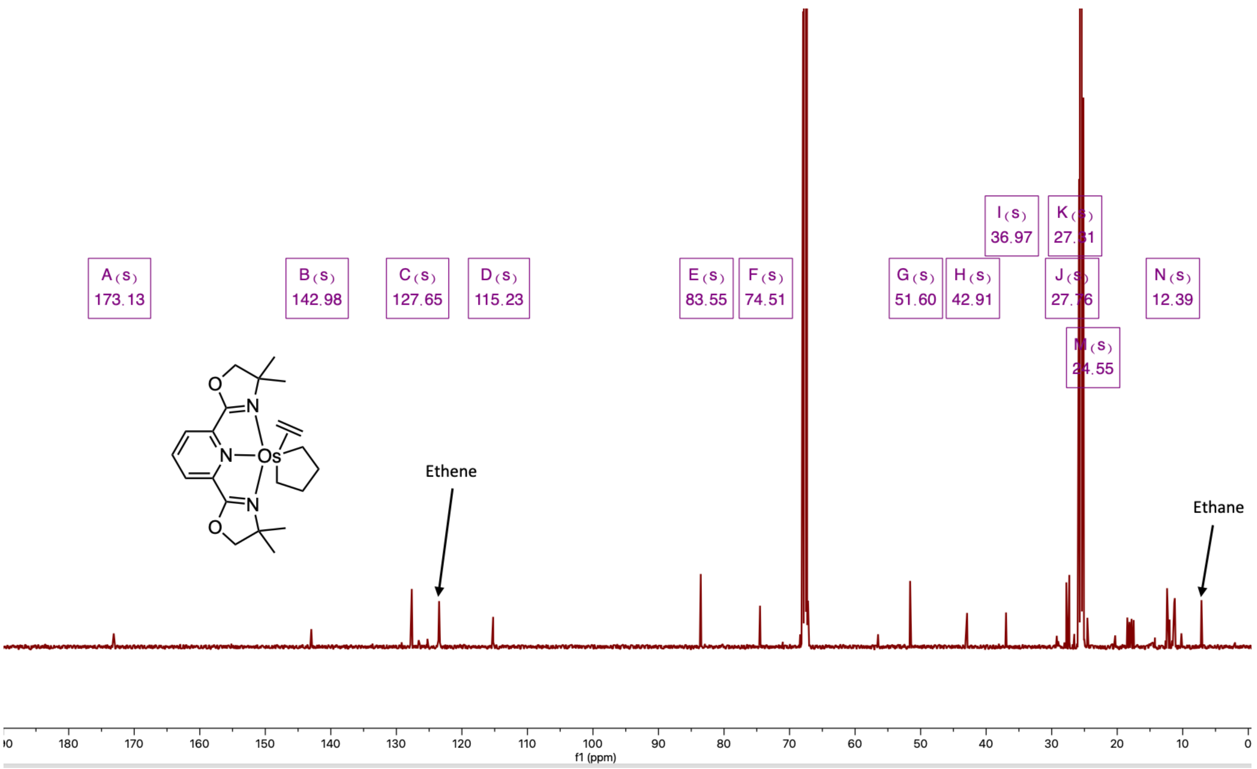

Figure S9. ^{13}C NMR spectrum of reaction of [(^{dm}Pybox)OsH_3]Na ([**1-H$_3$**]Na) with ethylene generating **2-C_2H_4** [(^{dm}Pybox)Os(cy-C_4H_8)(C_2H_4) in THF-d_8 at 298 K.

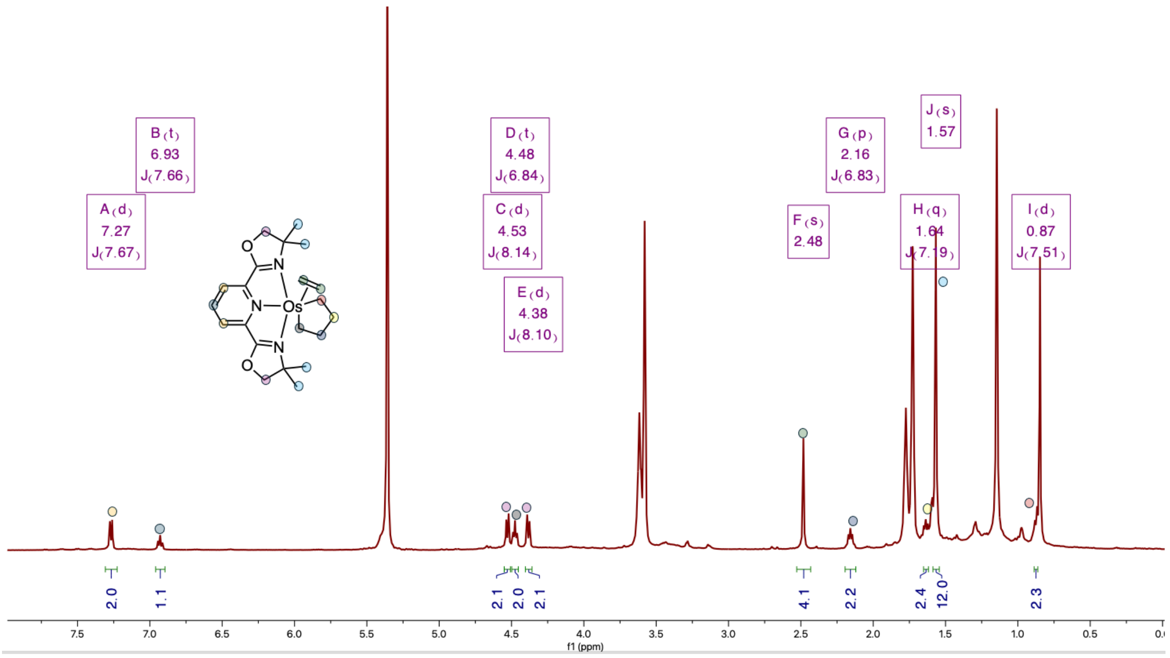

Figure S10. ^{1}H NMR spectrum of reaction of [(^{dm}Pybox)OsH_3]K ([**1-H$_3$**]K) with ethylene generating **2-C_2H_4** [(^{dm}Pybox)Os(cy-C_4H_8)(C_2H_4) in THF-d_8 at 298 K.

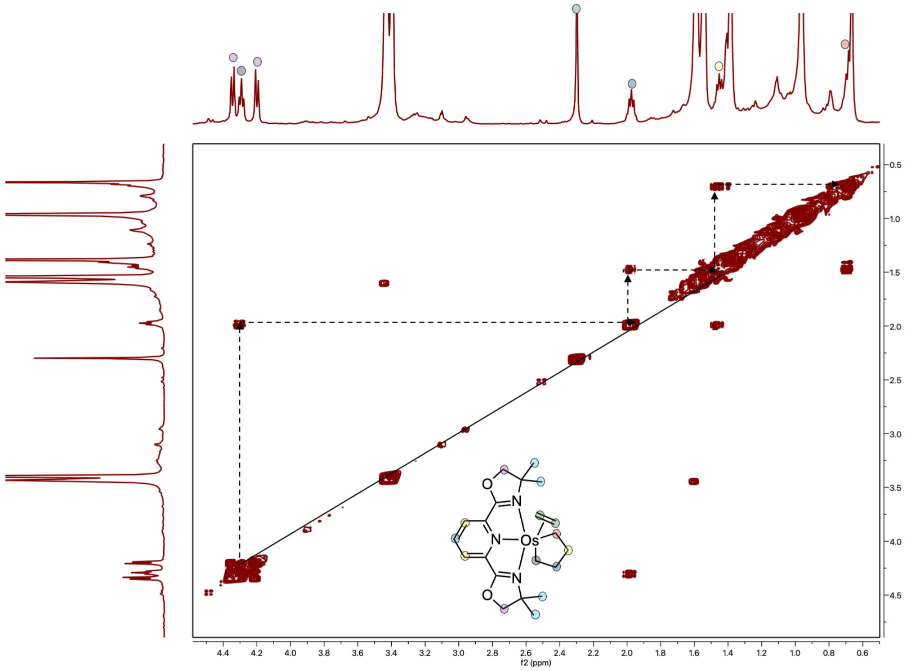

Figure S11. ^{1}H-^{1}H COSY NMR spectrum of reaction of [(^{dm}Pybox)OsH_3]K ([**1-H₃**]K) with ethylene generating **2-C_2H_4** [(^{dm}Pybox)Os(cy-C_4H_8)(C_2H_4) in THF-d_8 at 298 K.

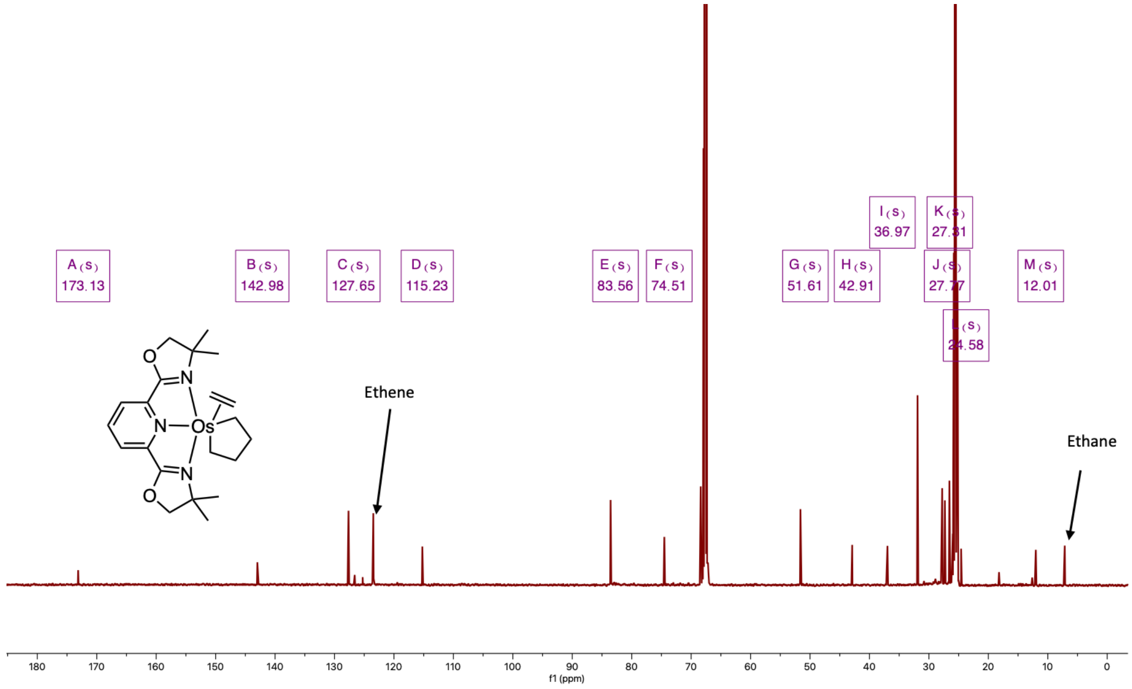

Figure S12. ^{13}C NMR spectrum of reaction of [(^{dm}Pybox)OsH_3]K ([**1-H₃**]K) with ethylene generating **2-C_2H_4** [(^{dm}Pybox)Os(cy-C_4H_8)(C_2H_4) in THF-d_8 at 298 K.

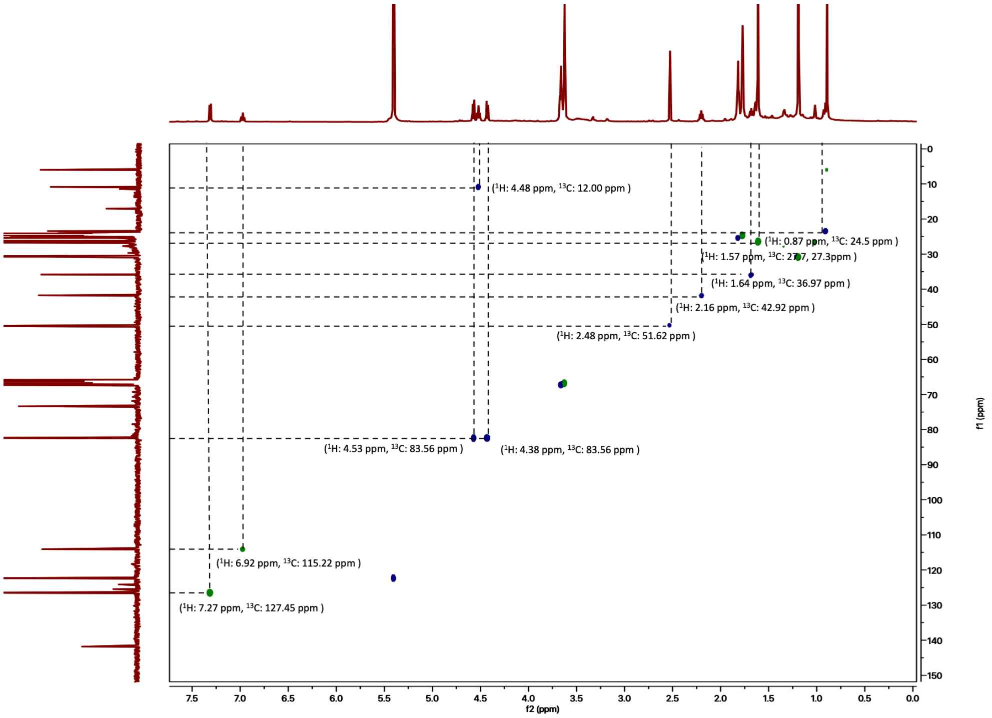

Figure S13. ^{1}H-^{13}C gHSQC NMR spectrum of reaction of [(^{dm}Pybox)OsH$_3$]K ([**1-H$_3$**]K) with ethylene generating **2-C$_2$H$_4$** [(^{dm}Pybox)Os(cy-C$_4$H$_8$)(C$_2$H$_4$) in THF-d$_8$ at 298 K. The x-axis is ^{1}H NMR and y-axis is ^{13}C NMR of the **2-C$_2$H$_4$.**

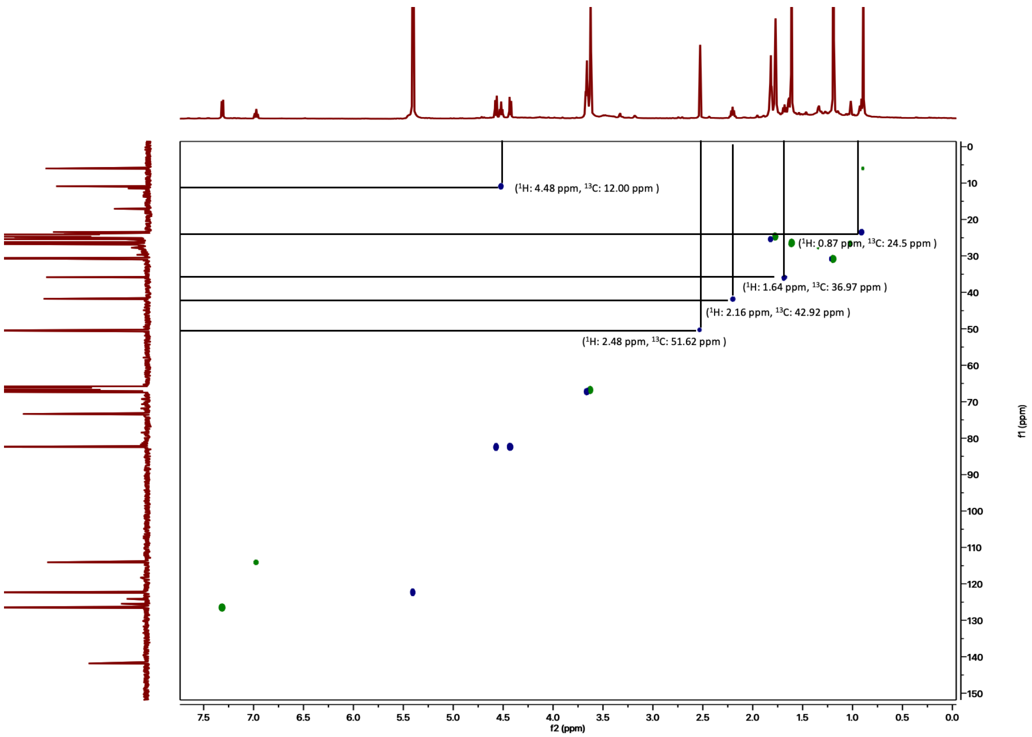

Figure S14. ^{1}H-^{13}C gHSQC NMR spectrum (emphasis on osmacyclopentane and bound ethylene) of reaction of [(^{dm}Pybox)OsH$_3$]K ([**1-H$_3$**]K)) with ethylene, generating (^{dm}Pybox)Os(cy-C$_4$H$_8$)(C$_2$H$_4$) (**2-C$_2$H$_4$**) in THF-d$_8$ at 298 K. The x-axis is ^{1}H NMR and y-axis is ^{13}C NMR of the **2-C$_2$H$_4$.**

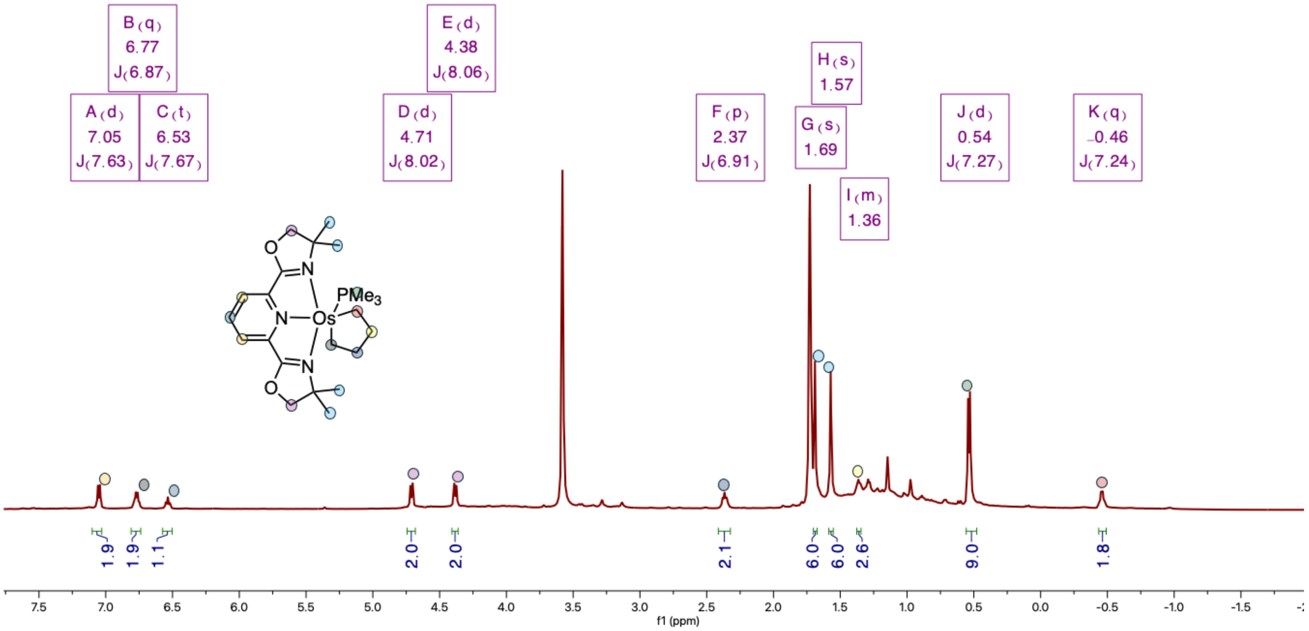

Figure S15. ^{1}H NMR spectrum of (^{dm}Pybox)Os(cy-C_4H_8)(PMe_3) (**2-PMe$_3$**) in THF-d_8 at 298 K.

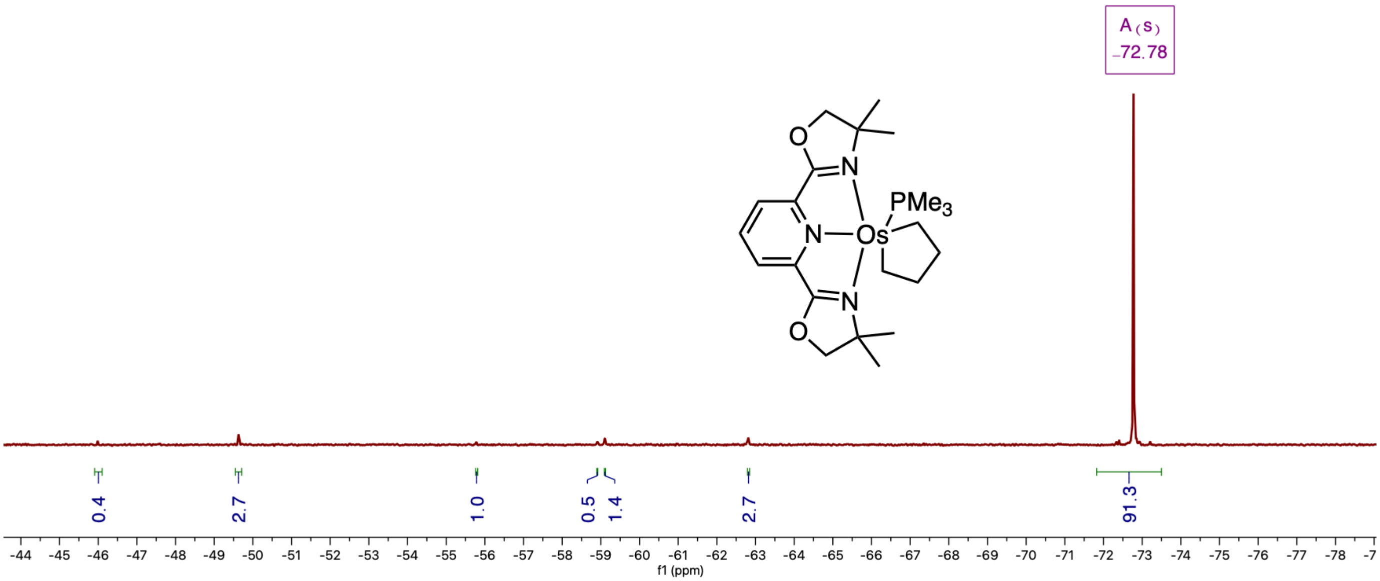

Figure S16. $^{31}P\{^{1}H\}$ NMR spectrum of (^{dm}Pybox)Os(cy-C_4H_8)(PMe_3) (**2-PMe$_3$**) in THF-d_8 at 298 K.

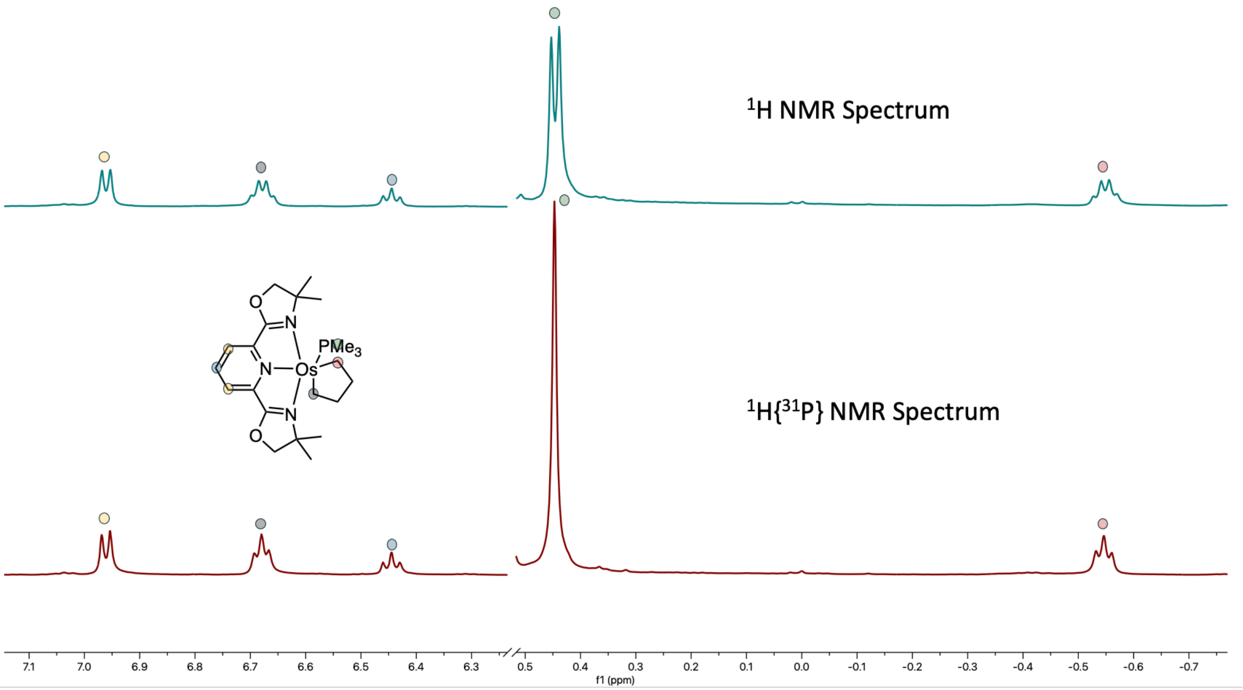

Figure S17. ^{31}P NMR spectrum of (^{dm}Pybox)Os(cy-C_4H_8)(PMe_3) (**2-PMe₃**) in THF-d_8 at 298 K.

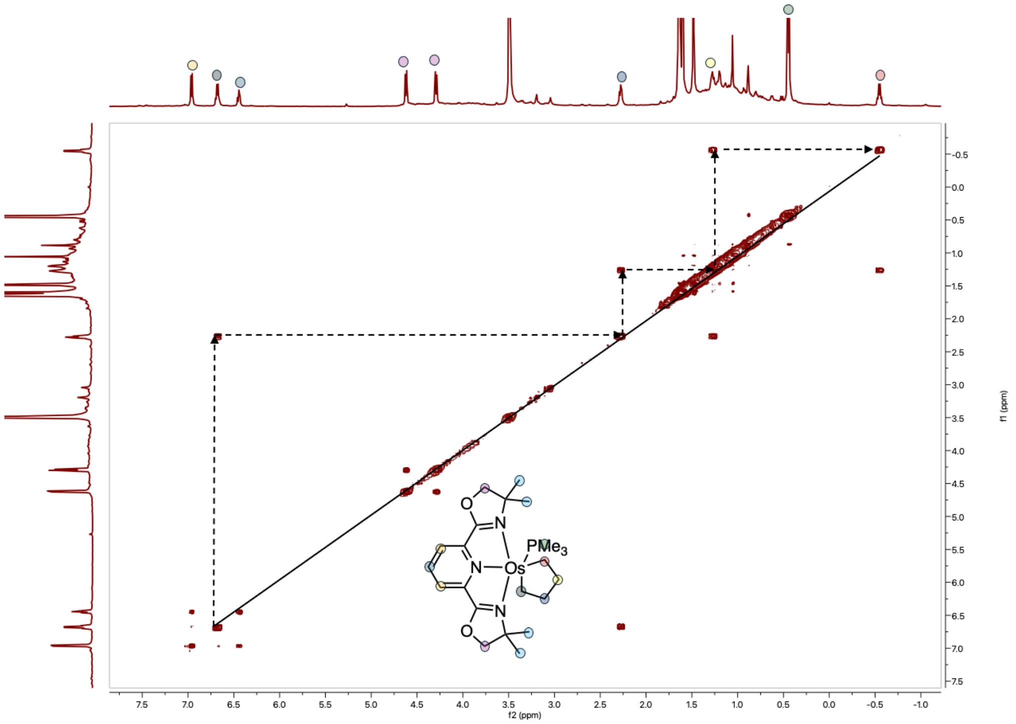

Figure S18. ^{1}H-^{1}H COSY NMR spectrum of **PMe₃** [(^{dm}Pybox)Os(cy-C_4H_8)(PMe_3) in THF-d_8 at 298 K.

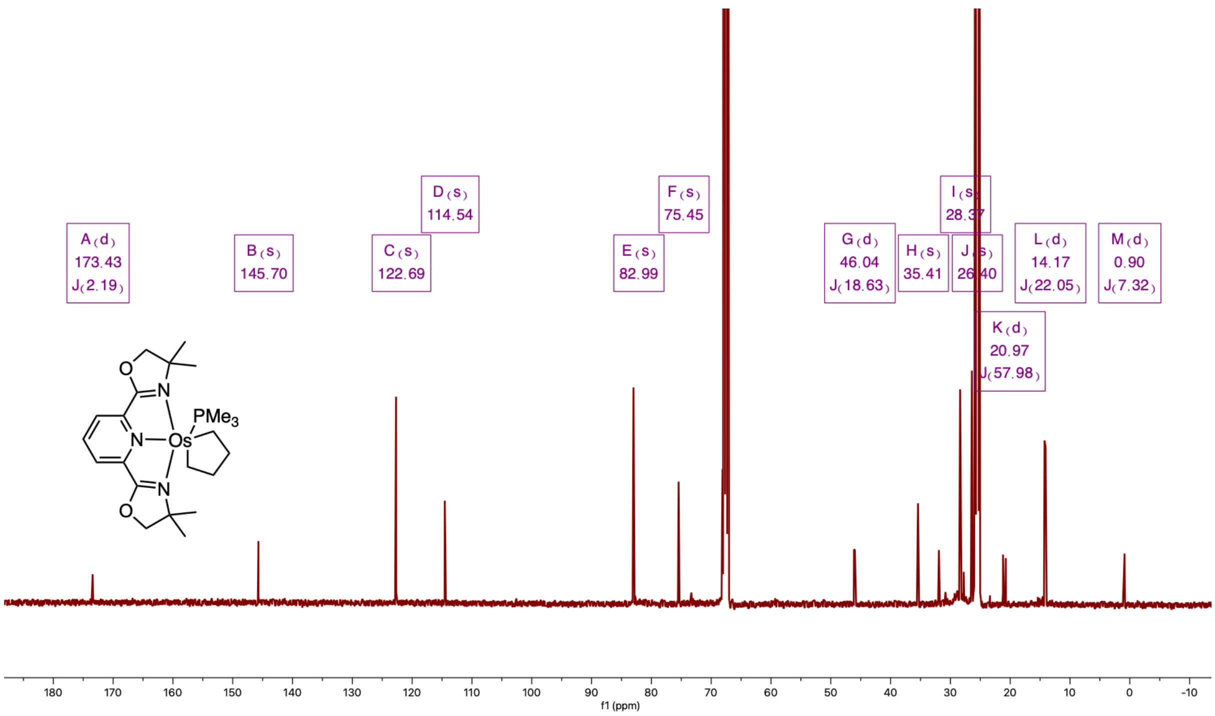

Figure S19. ^{13}C NMR spectrum of (^{dm}Pybox)Os(cy-C_4H_8)(PMe_3) (**2-PMe₃**) in THF-d_8 at 298 K.

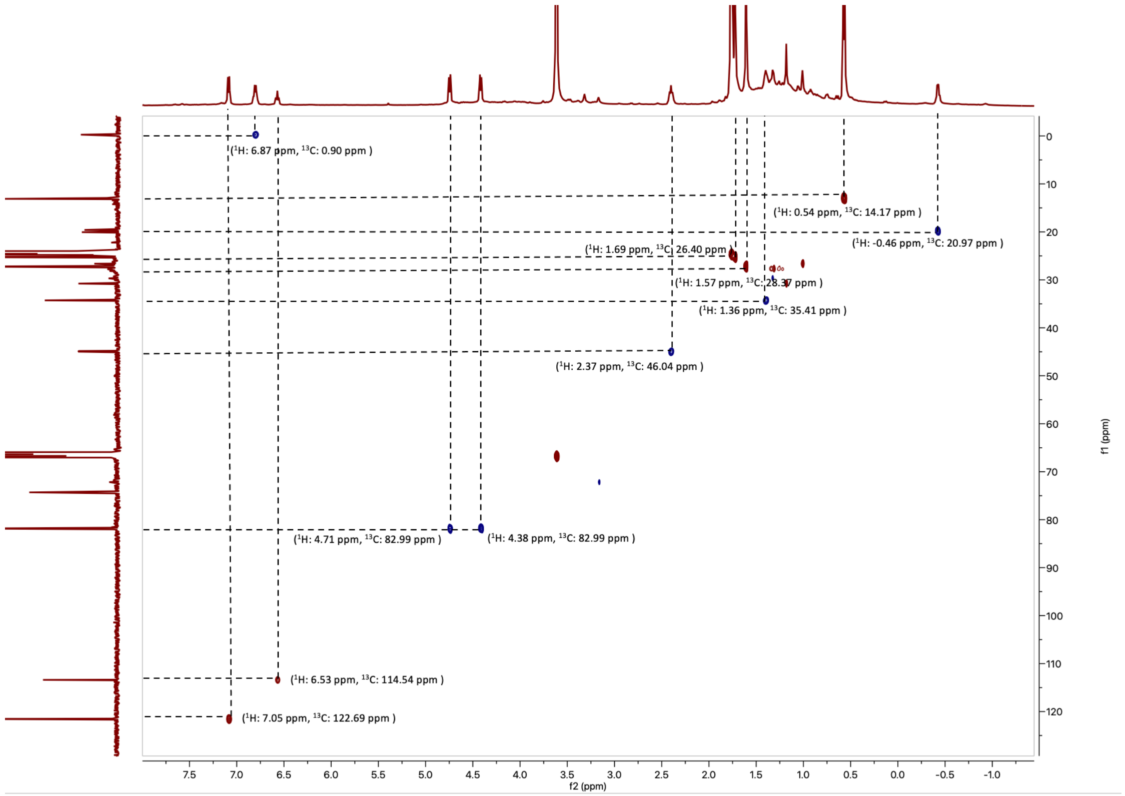

Figure S20. ^1H-^{13}C gHSQC NMR spectrum of (^{dm}Pybox)Os(cy-C_4H_8)(PMe_3) (**2-PMe₃**) in THF-d_8 at 298 K. The x-axis is ^1H NMR and y-axis is ^{13}C NMR of the **2-PMe₃**

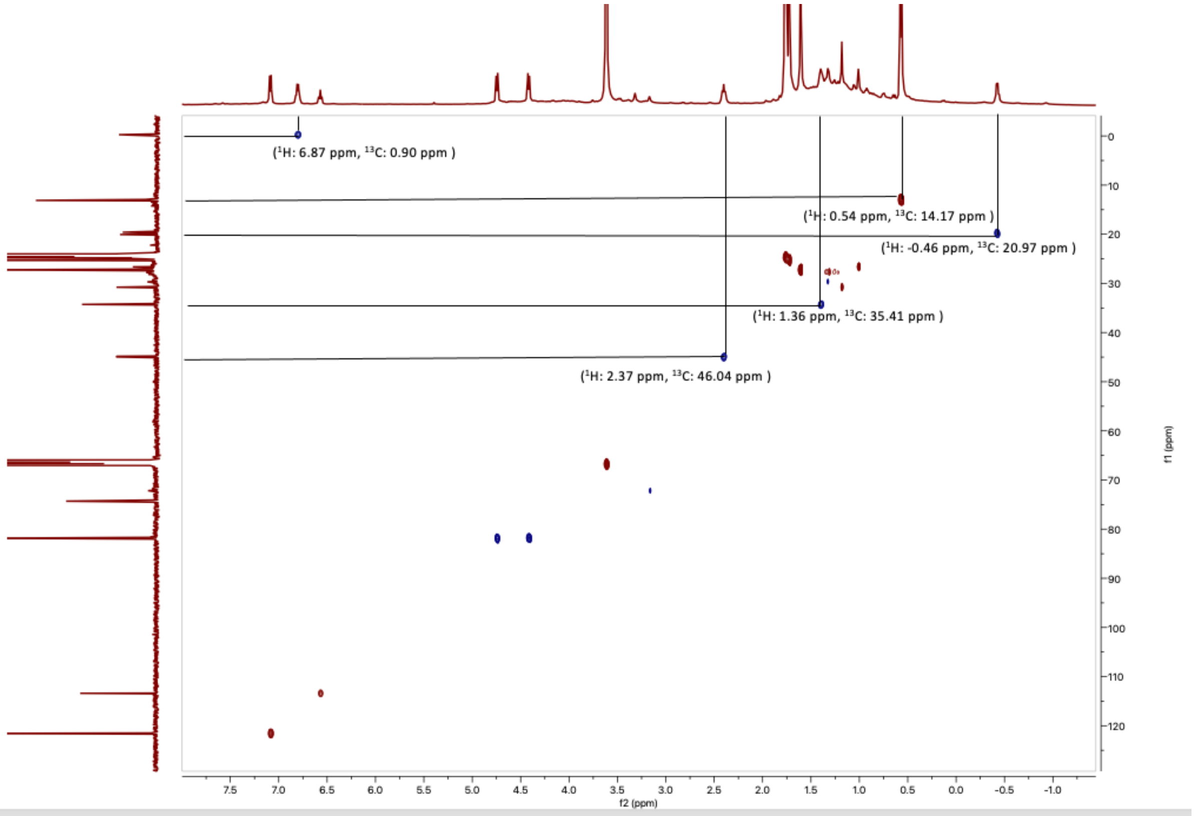

Figure S21. ^{1}H-^{13}C gHSQC NMR spectrum of (^{dm}Pybox)Os(cy-C_4H_8)(PMe_3) (**2-PMe$_3$**) in THF-d_8 at 298 K. The x-axis is ^{1}H NMR and y-axis is ^{13}C NMR of the **2-PMe$_3$.** (Emphasis on osmacyclopentane and bound PMe_3).

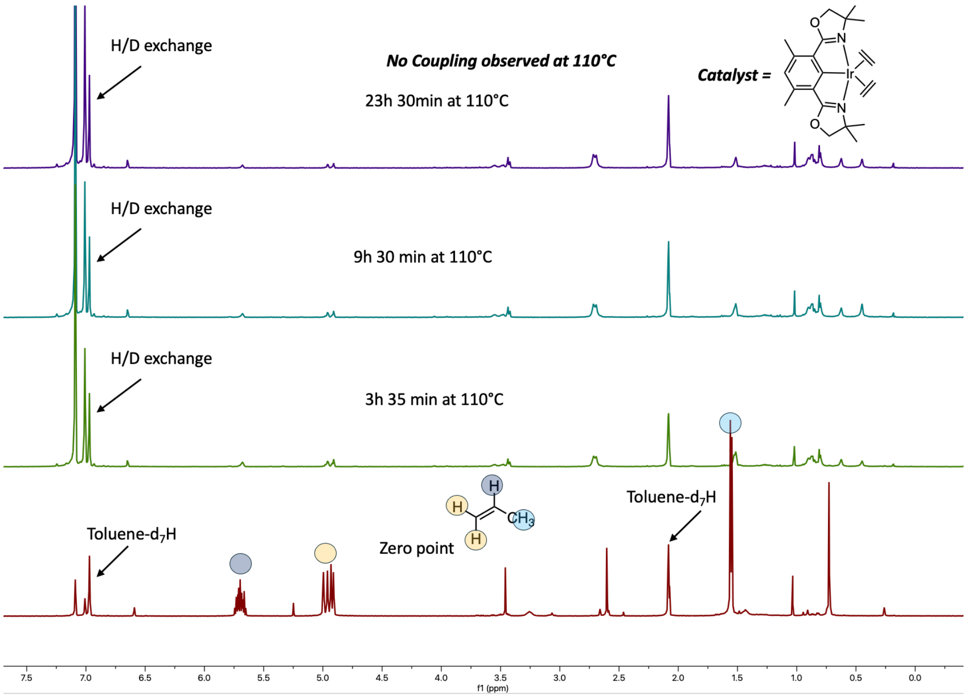

Figure S22. Reaction of propene with (Phebox)Ir(C_2H_4)$_2$ at 110 °C leading to H/D exchange and no dimerization.

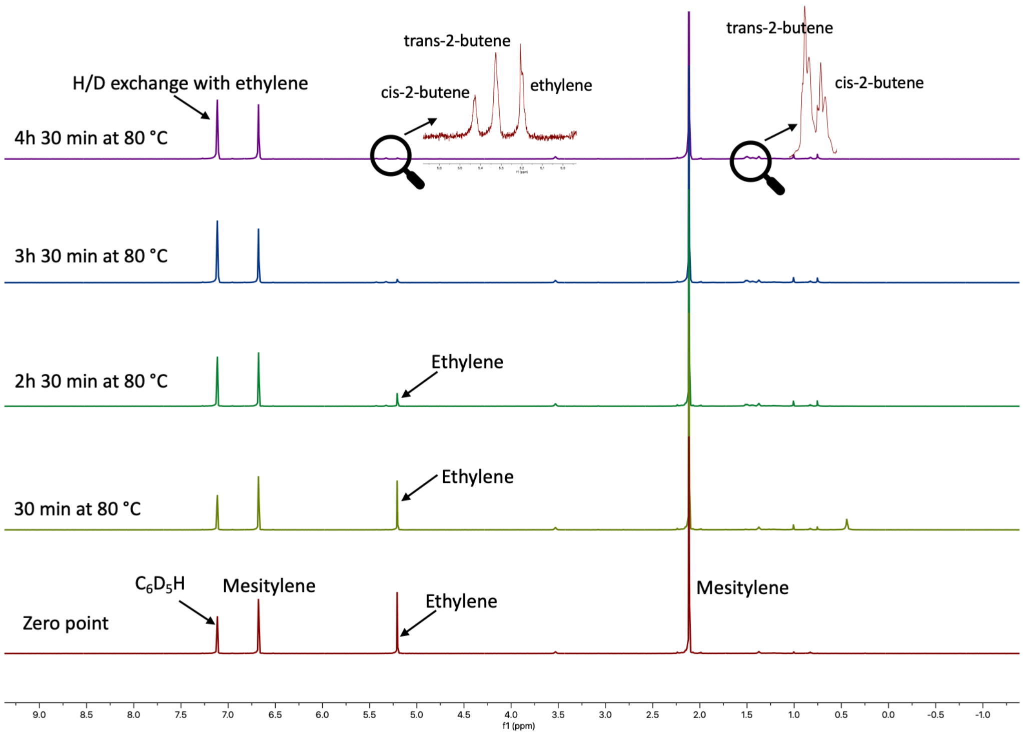

Figure S23. Reaction of 0.5 atm ethylene with **1-H_3^-** catalyst at 80°C leading to major dimerization products trans-2-butene and cis-2-butene along with H/D exchange with of benzene-d_6 with ethylene and product.

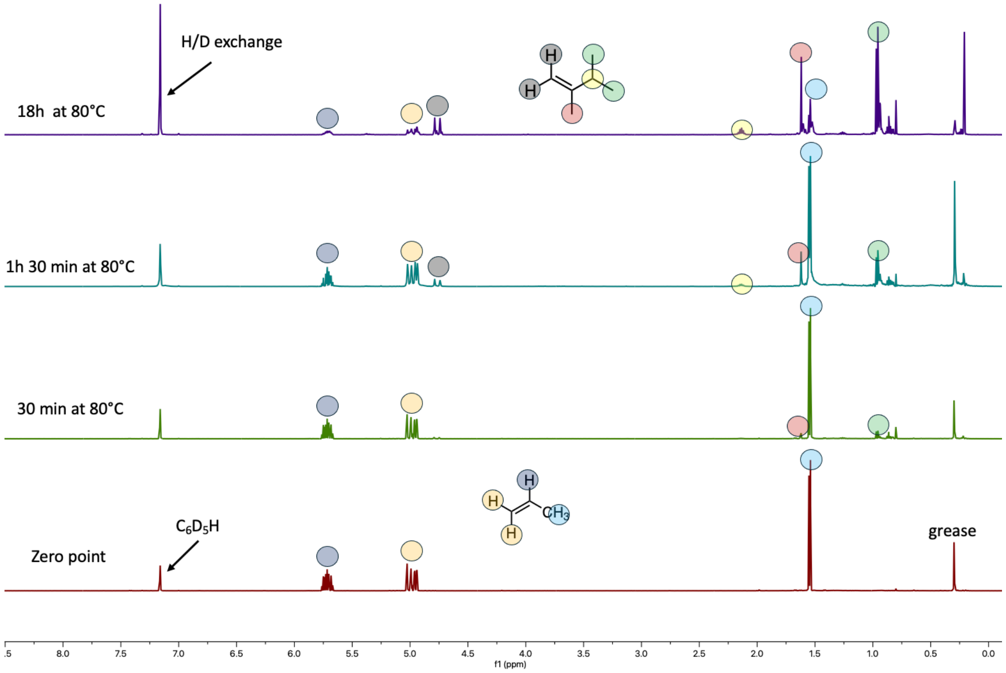

Figure S24. Reaction of propylene (1 atm) with **1-H_3^-** at 80 °C leading to major dimerization products 2,3-dimethylbutene along with H/D exchange of benzene-d_6 with propylene and product.

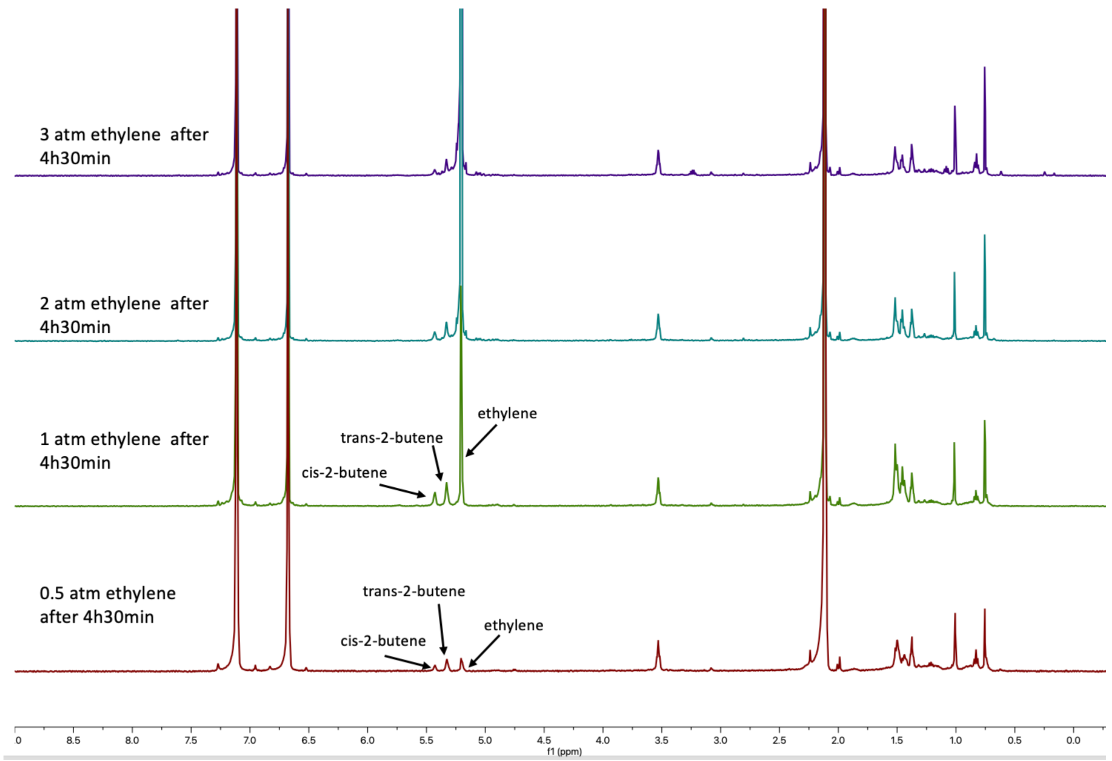

Figure S25. Reaction of ethylene (0.5 to 3 atm) with **1-H$_3^-$** at 80°C illustrating a negative order dependence with ethylene pressure.

S4. **Crystallographic Data**

S4.a. Crystallographic data for ^{dm}Pybox

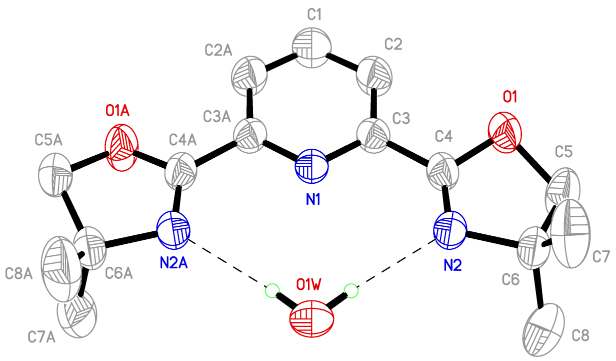

Figure S26. ORTEP representation (50% probability ellipsoids) of (^{dm}Pybox)-ligand determined by SDXRD. Hydrogen atoms other than those of water are omitted for clarity.

Identification code	SC_pybox	
Empirical formula	C22.03 H37.86 N3 O3	
Formula weight	392.73	
Temperature	293(2) K	
Wavelength	0.71073 Å	
Crystal system	Tetragonal	
Space group	$P4_2/ncm$	
Unit cell dimensions	a = 10.9346(16) Å	α = 90°
	b = 10.9346(16) Å	β = 90°
	c = 18.443(4) Å	γ = 90°
Volume	2205.2(8) $Å^3$	
Z	4	
Density (calculated)	1.183 Mg/m^3	
Absorption coefficient	0.079 mm^{-1}	
F(000)	860	
Crystal size	0.225 x 0.129 x 0.070 mm^3	
Theta range for data collection	2.209 to 25.343°.	
Index ranges	-11<=h<=13, -13<=k<=13, -22<=l<=14	
Reflections collected	11931	
Independent reflections	1098 [R(int) = 0.0199]	
Completeness to theta = 25.242°	100.0 %	
Absorption correction	Gaussian	
Max. and min. transmission	1.000 and 0.543	
Refinement method	Full-matrix least-squares on F^2	
Data / restraints / parameters	1098 / 263 / 132	
Goodness-of-fit on F^2	1.075	
Final R indices [I>2sigma(I)]	R1 = 0.0563, wR2 = 0.1652	
R indices (all data)	R1 = 0.0581, wR2 = 0.1676	
Extinction coefficient	n/a	
Largest diff. peak and hole	0.165 and -0.297 e.$Å^{-3}$	

S4.b. Crystallographic data for (^{dm}Pybox)$OsCl_3$ (1-Cl_3)

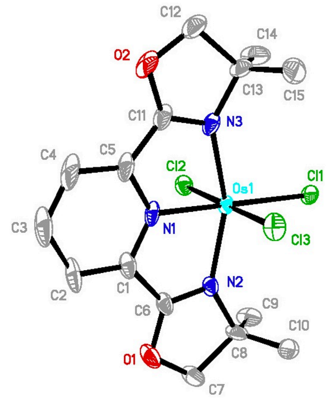

Figure S27. ORTEP representation (50% probability ellipsoids) of (^{dm}Pybox)$OsCl_3$ **(1-Cl_3)** determined by SDXRD. Hydrogen atoms and dichloromethane solvate are omitted for clarity.

Identification code	AP_141_P21n	
Empirical formula	C16 H21 Cl5 N3 O2 Os	
Formula weight	654.81	
Temperature	120(2) K	
Wavelength	0.71073 Å	
Crystal system	Monoclinic	
Space group	$P2_1/n$	
Unit cell dimensions	a = 13.311(3) Å	α = 90°
	b = 11.334(2) Å	β = 92.729(3)°
	c = 14.467(3) Å	γ = 90°
Volume	2180.1(7) $Å^3$	
Z	4	
Density (calculated)	1.995 Mg/m^3	
Absorption coefficient	6.479 mm^{-1}	
F(000)	1260	
Crystal size	0.31 x 0.23 x 0.01 mm^3	
Theta range for data collection	2.031 to 30.507°.	
Index ranges	-19<=h<=19, -16<=k<=16, -20<=l<=20	
Reflections collected	24382	
Independent reflections	6633 [R(int) = 0.0716]	
Completeness to theta = 25.242°	99.8 %	
Absorption correction	Semi-empirical from equivalents	
Max. and min. transmission	0.7464 and 0.5326	
Refinement method	Full-matrix least-squares on F^2	
Data / restraints / parameters	6633 / 464 / 270	
Goodness-of-fit on F^2	1.059	
Final R indices [I>2sigma(I)]	R1 = 0.0539, wR2 = 0.1110	
R indices (all data)	R1 = 0.0744, wR2 = 0.1191	
Extinction coefficient	n/a	
Largest diff. peak and hole	2.652 and -3.032 e.$Å^{-3}$	

S4.c. Crystallographic data for [(^{dm}Pybox)OsH$_3$][K(THF)$_2$] ([1-H$_3$]K(THF)$_2$

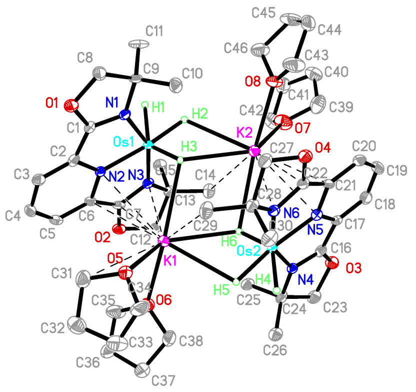

Figure S28. ORTEP representation (50% probability ellipsoids) of [(^{dm}Pybox)OsH$_3$][K(THF)$_2$] **([1-H$_3$]K(THF)$_2$** determined by SDXRD. Hydrogen atoms except for the hydride ligand are omitted for clarity.

Identification code	AP_Pybox-Os-K-THF_faces	
Empirical formula	C46 H75 K2 N6 O8 Os2	
Formula weight	1298.72	
Temperature	100(2) K	
Wavelength	1.54184 Å	
Crystal system	Monoclinic	
Space group	P2$_1$	
Unit cell dimensions	a = 11.34410(10) Å	α = 90°
	b = 19.42620(10) Å	β = 104.5970(10)°
	c = 11.92180(10) Å	γ = 90°
Volume	2542.44(4) Å^3	
Z	2	
Density (calculated)	1.696 Mg/m^3	
Absorption coefficient	11.206 mm^{-1}	
F(000)	1294	
Crystal size	0.130 x 0.090 x 0.060 mm^3	
Theta range for data collection	3.831 to 74.457°.	
Index ranges	-11<=h<=14, -23<=k<=24, -14<=l<=14	
Reflections collected	26296	
Independent reflections	9127 [R(int) = 0.0234]	
Completeness to theta = 67.684°	99.8 %	
Absorption correction	Gaussian	
Max. and min. transmission	1.000 and 0.744	
Refinement method	Full-matrix least-squares on F^2	
Data / restraints / parameters	9127 / 1 / 611	
Goodness-of-fit on F^2	1.058	
Final R indices [I>2sigma(I)]	R1 = 0.0183, wR2 = 0.0492	
R indices (all data)	R1 = 0.0185, wR2 = 0.0493	
Absolute structure parameter	0.496(8)	
Extinction coefficient	0.00012(2)	
Largest diff. peak and hole	0.726 and -0.809 e.Å^{-3}	

Explanation of the location of hydride ligands in the $[(^{dm}\text{Pybox})\text{OsH}_3][\text{K(THF)}_2]$

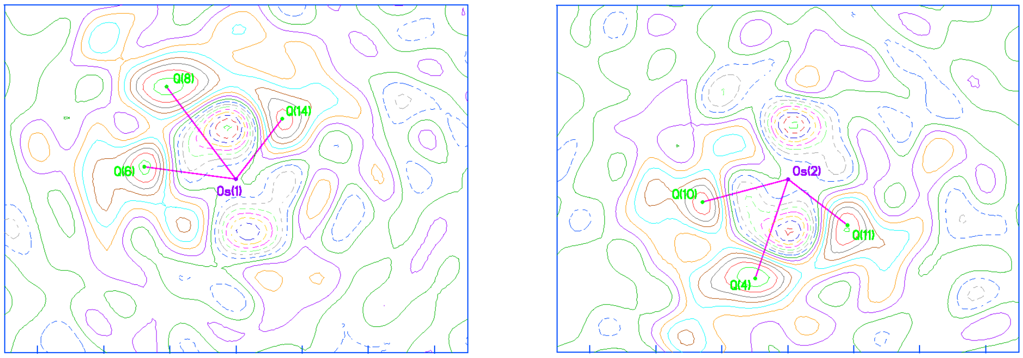

Figure S29. Residual electron density map of the plane containing hydride ligands in $[(^{dm}\text{Pybox})\text{OsH}_3][\text{K(THF)}_2]$ ([**1-H$_3$**][K(THF)$_2$]).

The relatively high level of confidence in locating all hydrido positions around the osmium atoms is based upon the [F(obs)-F(calc)] difference Fourier (or, equivalently, the difference electron density) calculation before inclusion of the hydrido atoms. The use of electron density maps to locate or confirm atom positions of relatively low Z in a crystallographic result is a fundamental tool with reliability based upon contour levels above background level. The maps attached here were calculated using the XP application in Sheldrick's program SHELXTL[S5, 6] and are contoured at approximately 1*sigma levels of the difference Fourier calculation, or about 1 e-/A^3 per level. The observed positive density (solid contours) has clear peak centroids on the maps and are denoted by peaks labeled Q6, Q8, Q14 about Os(1), which correspond to hydrido atoms H1, H2, H3, respectively, and by peaks Q10, Q4, Q11 about Os(2), which correspond to hydrido atoms H4, H5, H6, respectively. These hydrido atom positions did not change significantly upon refinement nor upon final refinements using Os-H bond distances that were restrained to the reliable average neutron diffraction result of 1.65 Å for terminal Os-H bonds.[S7-10] Thus, the final refined positions of these 6 hydrido atoms converged to approximately the same places as indicated by the labeled peaks in these two electron density maps.

S5. Kinetic Studies

To determine the overall rate equation for ethylene dimerization, the rate of formation of butenes was measured as a function of catalyst concentration and olefin pressure.

S5.a. Rate of reaction with varying ethylene pressure.

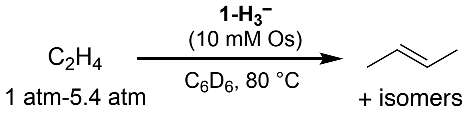

To measure the dependence of reaction rate on ethylene pressure, the concentration of the catalyst was kept constant, and ethylene pressure was varied from 1 atm to 5.4 atm (**Table S1**). The results show a negative order dependence on the ethylene pressure (order = -1.2; **Figure S30**).

Table S1. Dependence of reaction rate on pressure of ethylene.

**ln(P) (ethylene)**	0.0488	0.658	1.015	1.690
**ln(rate)**	-1.427	-2.007	-2.673	-3.411

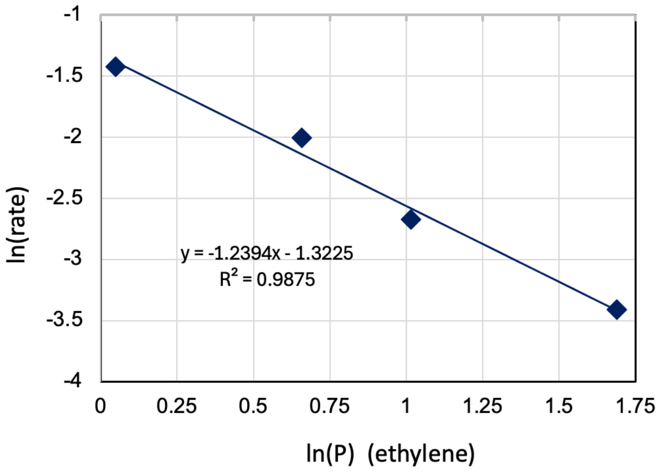

Figure S30. Rate of ethylene dimerization versus ethylene pressure.

S5.b. Rate of the reaction with catalyst concentration.

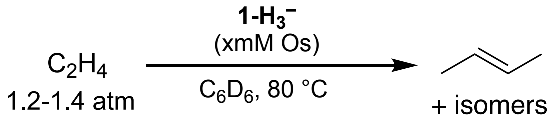

To measure the dependence of reaction rate on catalyst concentration, the pressure of ethylene was kept constant, and the concentration of catalyst was varied from 6 mM to 33 mM (**Table S2**). The results show an approximately first order dependence on the catalyst concentration (order = 1.26; **Figure S31**).

Table S2. Dependence of reaction rate on catalyst concentration.

**ln[catalyst]**	-4.995	-4.448	-4.097	-3.404	-3.867
**ln(rate)**	-2.333	-1.733	-1.362	-0.397	-0.794

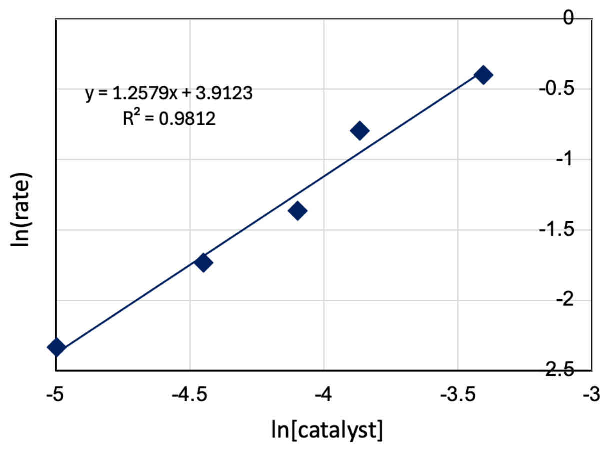

Figure S31. Rate of ethylene dimerization versus catalyst concentration.

S6. Computational Data

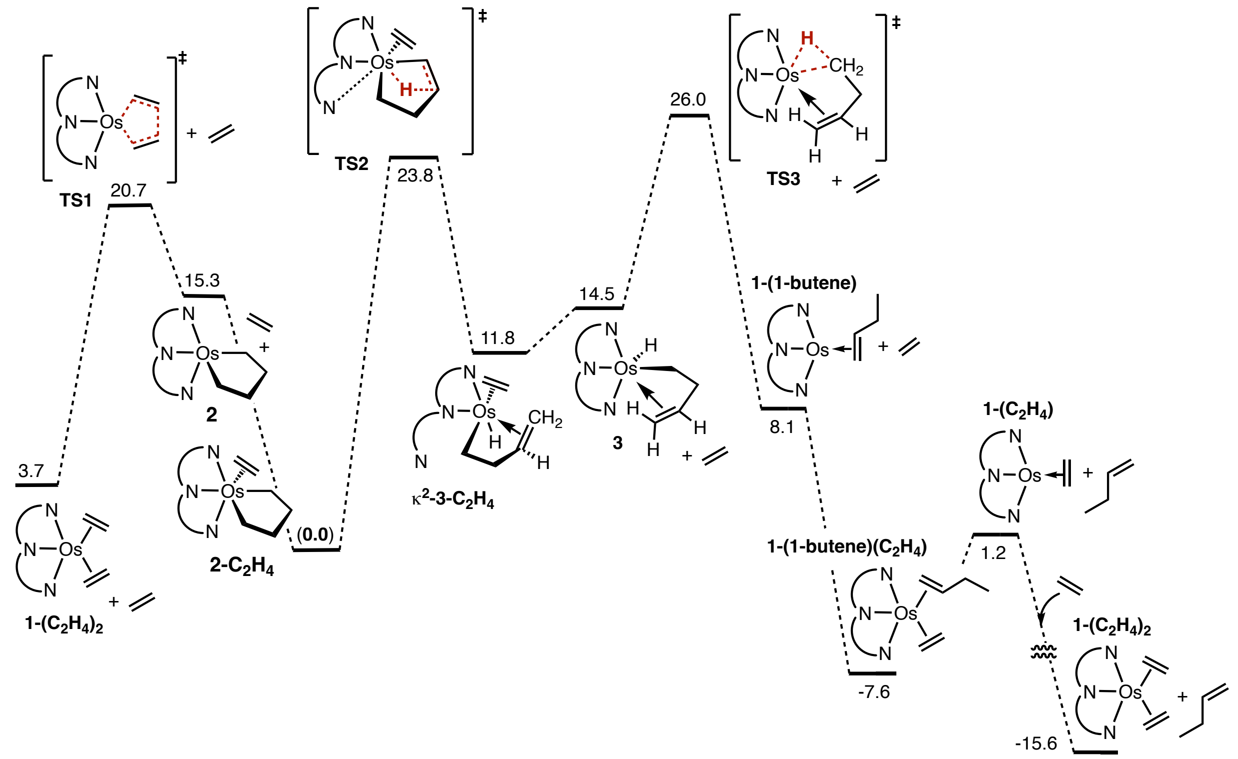

Figure S32. Mechanism for ethylene dimerization using **1**

Table S3. Computed Gibbs free energy of minima and TSs on different reaction pathways for ethylene dimerization into 1-butene by the osmium catalyst using different density functionals. Values are given in kcal/mol relative to the octahedral **2**-C_2H_4 complex.

	M06	wB97XD	B3LYP-D3BJ	PBE0-D3BJ
**1-$(C_2H_2)_2$ + 2 C_2H_4**	3.7	6.6	3.5	8.1
**TS1 + 2 C_2H_4**	20.7	20.6	19.2	22.1
**2 + 2 C_2H_4**	15.3	18.0	11.5	17.6
**2-C_2H_4 + C_2H_4**	0.0	0.0	0.0	0.0
**TS2 + C_2H_4**	23.8	23.7	26.0	23.8
**κ^2-3-(C_2H_4) + C_2H_4**	11.8	11.5	13.7	13.5
**3 + 2 C_2H_4**	14.5	14.1	11.3	15.4
**TS3 + 2 C_2H_4**	26.0	27.6	25.2	26.5
**1-(1-butene) + 2 C_2H_4**	8.1	17.8	8.4	17.9
**1-(1-butene)(C_2H_4) + C_2H_4**	-7.6	-5.6	-5.0	-5.3
**1-(C_2H_4) + 1-butene + C_2H_4**	1.2	11.9	1.9	10.6
**1-$(C_2H_4)_2$ + 1-butene**	-15.6	-13.1	-13.2	-14.5

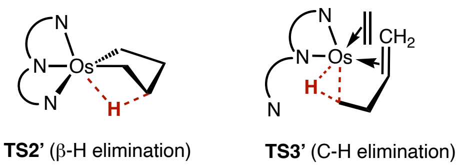

Figure S33. Alternate pathways for the butene formation

Table S4. Computed Gibbs free energy of alternate TSs for ethylene dimerization into 1-butene by the osmium catalyst using different density functionals. Values are given in kcal/mol relative to the octahedral **2**-C_2H_4 complex

Route to 1-butene prior to ethylene dissociation from **κ²-3**-(C_2H_4)				
	**M06**	**wB97XD**	**B3LYP-D3BJ**	**PBE0-D3BJ**
**TS3'**	29.4	31.3	33.6	31.3
Route to 1-butene following ethylene dissociation from **2**-(C_2H_4)				
	**M06**	**wB97XD**	**B3LYP-D3BJ**	**PBE0-D3BJ**
**TS2'**	37.7	39.2	37.3	39.2

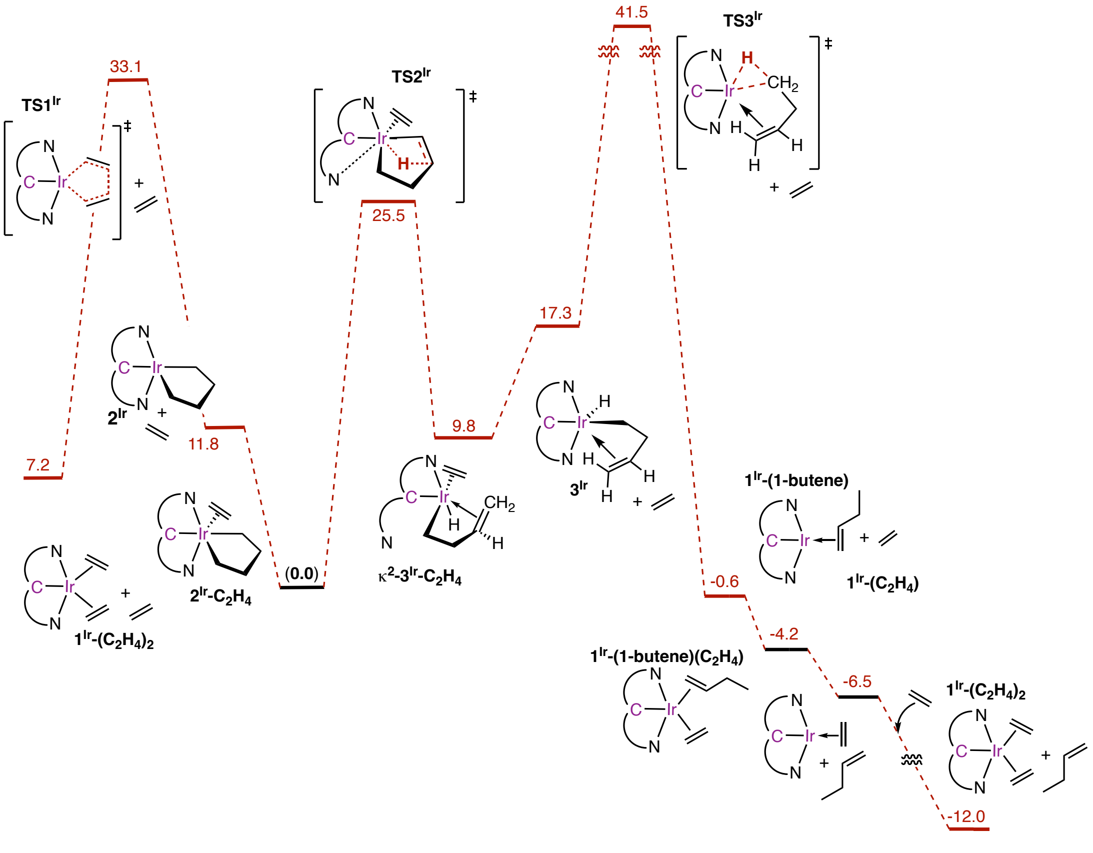

Figure S34. Mechanism for ethylene dimerization using $\mathbf{1^{Ir}}$.

Table S5. Computed Gibbs free energy of minima and TSs on different reaction pathways for ethylene dimerization into 1-butene by the Iridium catalyst using different density functionals. Values are given in kcal/mol relative to the octahedral **2**^{Ir}-C_2H_4 complex.

	M06	wB97XD	B3LYP-D3BJ	PBE0-D3BJ
**1^{Ir}-(C_2H_2)$_2$ + 2 C_2H_4**	7.2	9.2	6.3	11.2
**TS1^{Ir} + 2 C_2H_4**	33.1	36.6	32.7	36.8
**2^{Ir} + 2 C_2H_4**	11.8	13.7	10.4	16.3
**2^{Ir}-C_2H_4 + C_2H_4**	0.0	0.0	0.0	0.0
**TS2^{Ir} + C_2H_4**	25.5	26.0	27.5	25.6
**κ^2-3^{Ir}-(C_2H_4) + C_2H_4**	9.8	11.2	11.0	12.0
**3^{Ir} + 2 C_2H_4**	17.3	20.6	17.7	24.2
**TS3^{Ir} + 2 C_2H_4**	41.5	45.9	41.6	44.9
**1^{Ir}-(1-butene) + 2 C_2H_4**	-0.6	9.6	6.1	14.1
**1^{Ir}-(1-butene)(C_2H_4) + C_2H_4**	-4.2	-3.6	-2.3	-2.7
**1^{Ir}-(C_2H_4) + 1-butene + C_2H_4**	-6.5	2.8	-2.5	5.3
**1^{Ir}-(C_2H_4)$_2$ + 1-butene**	-12.0	-10.5	-10.4	-11.4

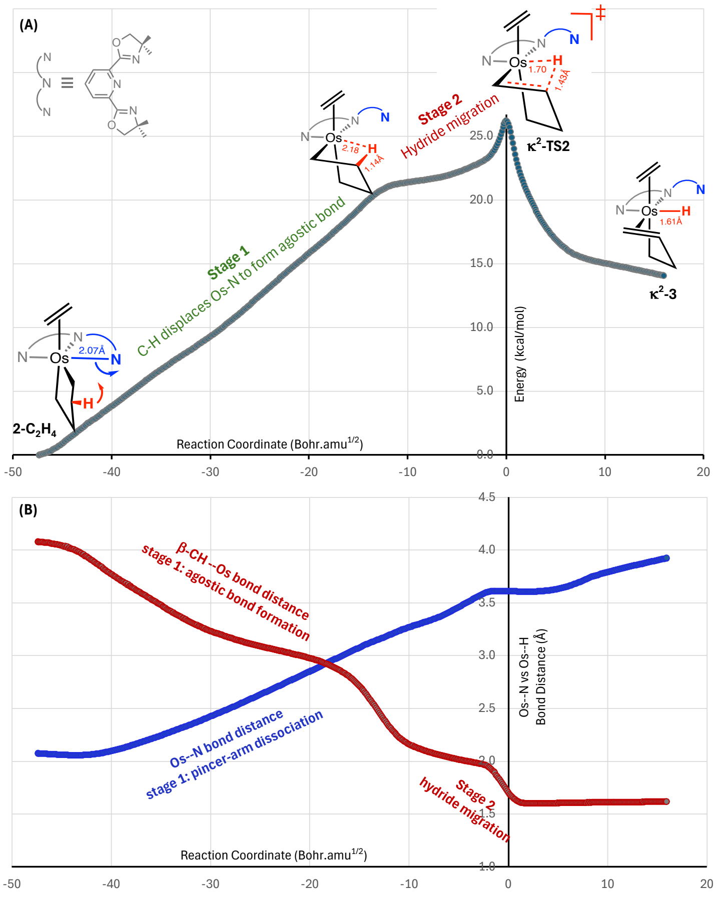

Figure S35. Intrinsic Reaction Coordinate (IRC) originating from **κ^2-TS2** revealing a mode in which the octahedral complex **2**-C_2H_2 undergoes asynchronous concerted κ^3-κ^2 dechelation of the Pybox ligand coupled to β-agostic bond formation and β-H migration. (**A**) Change of the energy along the reaction coordinate. (**B**) Change in the Os-N bond distance of the dissociating pincer-arm and the CH--Os bond distance undergoing reaction.

S7. References

S1. Lu, D.-F.; Zhu, C.-L.; Jia, Z.-X.; Xu, H., Iron(II)-Catalyzed Intermolecular Amino-Oxygenation of Olefins through the N–O Bond Cleavage of Functionalized Hydroxylamines *J. Am. Chem. Soc.* **2014,** *136*, 13186-13189.

S2. Nishiyama, H.; Sakaguchi, H.; Nakamura, T.; Horihata, M.; Kondo, M.; Itoh, K., Chiral and C2-symmetrical bis(oxazolinylpyridine)rhodium(III) complexes: effective catalysts for asymmetric hydrosilylation of ketones *Organometallics* **1989,** *8*, 846-848.

S3. Lease, N.; Pelczar, E. M.; Zhou, T.; Malakar, S.; Emge, T. J.; Hasanayn, F.; Krogh-Jespersen, K.; Goldman, A. S., PNP-Pincer Complexes of Osmium: Comparison with Isoelectronic (PCP)Ir and (PNP)Ir^+ Units *Organometallics* **2018,** *37*, 314-326.

S4. Gao, Y.; Emge, T. J.; Krogh-Jespersen, K.; Goldman, A. S., Selective Dehydrogenative Coupling of Ethylene to Butadiene via an Iridacyclopentane Complex *J. Am. Chem. Soc.* **2018,** *140*, 2260-2264.

S5. Sheldrick, G., SHELXTL v. 6.12, structure determination software suite. Bruker AXS, Madison, Wisconsin, USA: 2000.

S6. Sheldrick, G., A short history of SHELX *Acta Cryst. Section A* **2008,** *64*, 112-122.

S7. Groom, C. R.; Bruno, I. J.; Lightfoot, M. P.; Ward, S. C., The Cambridge Structural Database. *Acta Cryst. Section B* **2016,** *72*, 171-179.

S8. Hart, D. W.; Bau, R.; Koetzle, T. F., Neutron and x-ray diffraction studies on tris(dimethylphenylphosphine)osmium tetrahydride *J. Am. Chem. Soc.* **1977**, *99*, 7557-7564.

S9. Howard, J. A. K.; Johnson, O.; Koetzle, T. F.; Spencer, J. L., Crystal and molecular structure of bis(diisopropylphenylphosphine)hexahydridoosmium, $[OsH_6(PC_{12}H_{19})_2]$: single-crystal neutron diffraction study at 20 K *Inorg. Chem.* **1987,** *26*, 2930-2933.

S10. Johnson, T. J.; Albinati, A.; Koetzle, T. F.; Ricci, J.; Eisenstein, O.; Huffman, J. C.; Caulton, K. G., $OsH_5(PMe_2Ph)^{3+}$: Structure, Reactivity, and Its Use as a Catalyst Precursor for Olefin Hydrogenation and Hydroformylation *Inorg. Chem.* **1994,** *33*, 4966-4976.
